# Cancer-associated fibroblast-derived periostin promotes papillary thyroid tumor growth through integrin-FAK-STAT3 signaling

**DOI:** 10.7150/thno.94207

**Published:** 2024-05-13

**Authors:** Xiaorui Jin, Qianmei Deng, Shuting Ye, Shuting Liu, Yilong Fu, Yingfu Liu, Guoyang Wu, Gaoliang Ouyang, Tiantian Wu

**Affiliations:** 1 State Key Laboratory of Cellular Stress Biology, School of Life Sciences, Faculty of Medicine and Life Sciences, Xiamen University, Xiamen 361102, China.; 2 Department of Hematology, the First Affiliated Hospital of Xiamen University and Institute of Hematology, School of Medicine, Xiamen University, Xiamen 361003, China.; 3 Department of General Surgery, Zhongshan Hospital Affiliated to Xiamen University, Xiamen 361004, China.; 4 Department of Basic Medical Sciences, School of Medicine, Xiamen University, Xiamen 361102, China.

**Keywords:** periostin, extracellular matrix, papillary thyroid tumor, cancer-associated fibroblast, IL-4

## Abstract

**Background:** Periostin (POSTN) is a critical extracellular matrix protein in various tumor microenvironments. However, the function of POSTN in thyroid cancer progression remains largely unknown.

**Methods:** *Postn* and *Rag1* knock-out mice and orthotopic mouse models were used to determine the role of POSTN on papillary thyroid tumor progression. Immunofluorescence, cell co-culture, fluorescence *in situ* hybridization, chromatin immunoprecipitation assay, recombinant protein and inhibitor treatment were performed to explore the underlying mechanisms of POSTN-promoted papillary thyroid tumor growth.

**Results:** POSTN is up-regulated in papillary thyroid tumors and negatively correlates with the overall survival of patients with thyroid cancer. Cancer-associated fibroblast (CAF)-derived POSTN promotes papillary thyroid tumor growth *in vivo* and *in vitro*. POSTN deficiency in CAFs significantly impairs CAF-promoted papillary thyroid tumor growth. POSTN promotes papillary thyroid tumor cell proliferation and IL-4 expression through integrin-FAK-STAT3 signaling. In turn, tumor cell-derived IL-4 induces the activation of CAFs and stimulates POSTN expression by activating STAT6. We reveal the crucial role of CAF-derived POSTN and tumor cell-derived IL-4 in driving the development of papillary thyroid tumors through the POSTN-integrin-FAK-STAT3-IL-4 pathway in tumor cells and IL-4-STAT6-POSTN signaling in CAFs.

**Conclusion:** Our findings underscore the significance of POSTN and IL-4 as critical molecular mediators in the dynamic interplay between CAFs and tumor cells, ultimately supporting the growth of papillary thyroid tumors.

## Introduction

Thyroid cancer is the most prevalent endocrine cancer, accounting for approximately 3% of all cancer cases worldwide, with an estimated 586,000 new cases diagnosed in 2020 according to the International Agency for Research on Cancer 1, 2. The most common type of thyroid cancer is papillary thyroid cancer, making up roughly 80% of all cases 3, followed by follicular thyroid cancer, medullary thyroid cancer and anaplastic thyroid cancer. Among them, papillary thyroid cancer and follicular thyroid cancer are classified as differentiated thyroid cancer, both originating from the follicular epithelial cells. The overall prognosis for most papillary thyroid cancer patients is promising, with a 5-year survival rate exceeding 90% 4. However, a small subset of patients develops aggressive papillary thyroid cancer with local and distant metastases or recurrence, leading to an unsatisfactory outcome 4, 5. Therefore, investigating the underlying molecular mechanisms of papillary thyroid cancer progression is critical for the development of novel and effective therapies.

The tumor microenvironment (TME) is a complex entity composed of tumor cells surrounded by cancer-associated fibroblasts (CAFs), immune cells, endothelial cells, extracellular matrix (ECM), soluble factors, etc. 6, 7. Within the TME of thyroid cancer, CAFs remodel the ECM and generate a tumor-supportive niche, which plays a crucial role in thyroid cancer progression 8. POSTN is a matricellular protein and binds to both ECM and cell surface integrin receptors, thus playing a crucial role in mediating cell-cell and cell-matrix interactions 7, 9. Recent studies have demonstrated that POSTN is highly expressed in various tumor microenvironments and promotes tumor progression 9, 10. We previously demonstrated that bone marrow mesenchymal stromal cell-derived POSTN promotes leukemia cell proliferation via integrin-ILK-NF-κB pathway 11. During colorectal tumorigenesis, fibroblast-derived POSTN promotes colorectal tumor cell proliferation through integrin-FAK-Src-YAP pathway 12. Previous study demonstrated that POSTN is upregulated in papillary thyroid cancer 13. However, how POSTN regulates papillary thyroid cancer progression remains largely unclear.

In this study, we employed an orthotopic mouse model of papillary thyroid tumor to investigate the function of POSTN in papillary thyroid cancer. Our findings demonstrated that POSTN is highly expressed in papillary thyroid tumors and POSTN deficiency suppresses papillary thyroid tumor growth. We further elucidated that POSTN is mainly derived from activated CAFs in papillary thyroid tumor tissues and POSTN deficiency in CAFs impairs CAF-promoted papillary thyroid tumor growth *in vivo* and *in vitro*. Our results suggest that CAF-derived POSTN promotes the expression of IL-4 in papillary thyroid tumor cells through integrin-FAK-STAT3 pathway. Conversely, papillary thyroid tumor cell-derived IL-4 can increase CAF activation and the expression of POSTN in CAFs via STAT6 activation. Our findings suggest that the crosstalk between CAFs and tumor cells via POSTN and IL-4 plays an important role in papillary thyroid tumor progression.

## Results

### POSTN is highly expressed in papillary thyroid tumors and correlates with poor prognosis in patients with thyroid cancer

**Figure 1:**
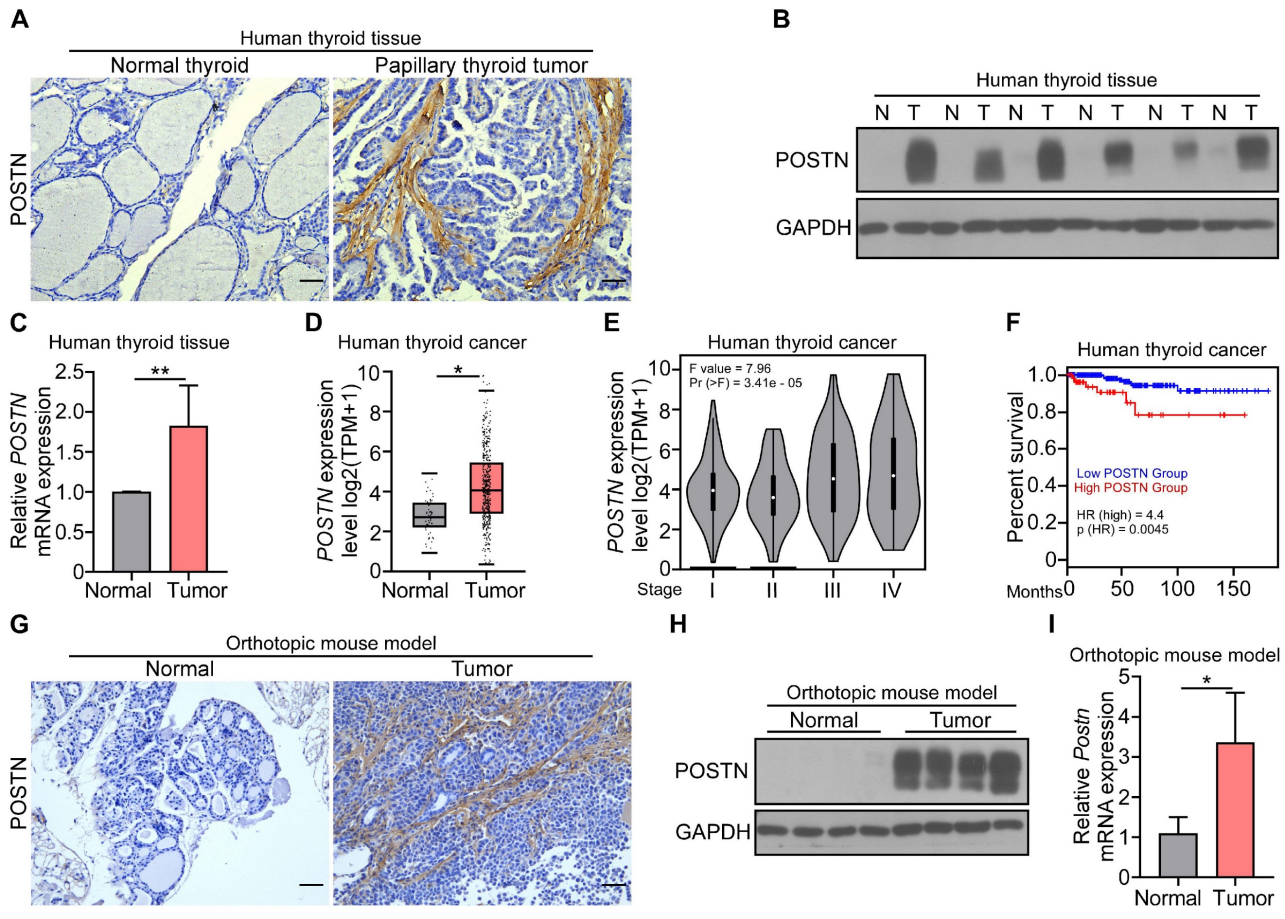
** Expression of POSTN in human and mouse papillary thyroid tumors and its clinical prevalence.** (**A**) Representative images of the immunohistochemical staining of POSTN in human papillary thyroid tumor and adjacent normal tissue. Scale bars, 50 μm. (**B** and **C**) Western blot (**B**) and qRT-PCR (**C**) analyses of POSTN in the tissues of human papillary thyroid tumors (T) and adjacent normal tissues (N) (Student's t test, n = 5). (**D**) GEPIA database analysis of POSTN expression in human normal thyroid (n = 59) and thyroid tumors (n = 512). Transcripts per million (TPM). Student's t test. (**E**) GEPIA database stage-plot analysis of the relationship between POSTN expression and tumor stages in thyroid cancer (χ^2^ test). (**F**) GEPIA database correlation analysis of overall survival with POSTN levels in thyroid cancer (Low POSTN group, n = 459; high POSTN group, n = 128, log rank test). (**G**-**I**) Immunohistochemical staining (**G**), western blot (**H**) and qRT-PCR (**I**) analyses of POSTN in thyroid tissues from mice after the orthotopic injection of IHH-4 cells or PBS (Student's t test, n = 3). Scale bars, 50 μm. Data are shown as means ± SEM. *, *P* < 0.05; **, *P* < 0.01.

To investigate the role of POSTN in thyroid cancer, we first determined the expression of POSTN in clinical samples. Immunohistochemical staining, western blot and qRT-PCR analyses collectively revealed a marked upregulation of POSTN in clinical papillary thyroid tumors compared to their matched adjacent normal tissues (**Figure 1A-C**). We further conducted an integrated analysis of 571 samples (59 normal thyroid, 512 thyroid tumors) from the GEPIA database and also found a significant increase of POSTN expression in human thyroid tumors relative to normal thyroid tissues (**Figure 1D**). Moreover, POSTN expression exhibited a positive correlation with advanced tumor stages (**Figure 1E**), and patients with high POSTN expression had a reduced overall survival (**Figure 1F**).

To further explore the function of POSTN in the tumorigenesis of papillary thyroid cancer, IHH-4 cells were orthotopically injected into the right thyroid gland of* Postn*^+/+^*Rag1*^-/-^ mice, mice were sacrificed after two weeks, and papillary thyroid tumor tissues were collected for further analysis. Immunohistochemical staining showed that POSTN was significantly elevated in tumor-bearing mice compared to control mice (**Figure 1G**). The upregulation of POSTN in papillary thyroid tumors of mice was further confirmed by western blot and qRT-PCR analyses (**Figure 1H-I**). Taken together, our results indicate that POSTN is significantly increased in papillary thyroid tumors and correlates with poor prognosis of patients with thyroid cancer.

### POSTN is mainly derived from CAFs in papillary thyroid tumors

**Figure 2:**
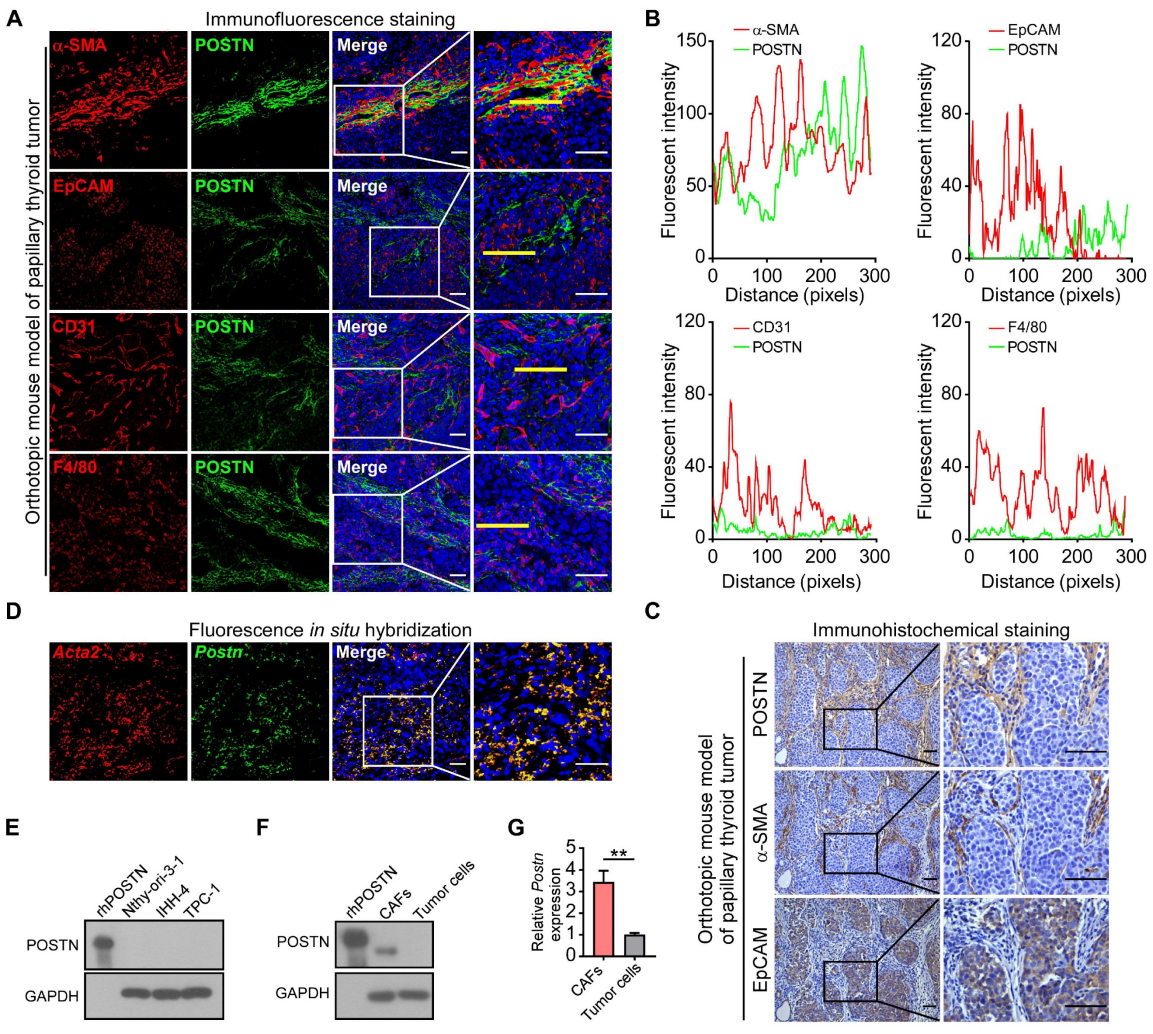
** POSTN is mainly derived from CAFs in mouse papillary thyroid tumors.** (**A**) Representative immunofluorescence co-staining of POSTN with α-SMA, EpCAM, CD31 or F4/80 in orthotopic mouse thyroid tumors. Nuclei were counterstained with DAPI. Scale bars, 50 μm. (**B**) Plots of fluorescent intensity along the yellow line of images in (**A**). (**C**) Representative immunohistochemical staining of POSTN, α-SMA and EpCAM in the orthotopic thyroid tumors. Scale bars, 50 μm. (**D**) Fluorescence *in situ* hybridization of *Postn* and *Acta2* in orthotopic thyroid tumors. Nuclei were counterstained with DAPI. Scale bars, 50 μm. (**E**) Western blot analysis of POSTN expression in Nthy-ori-3-1, IHH-4 and TPC-1 cells. rhPOSTN was used as a positive control. (**F** and **G**) POSTN protein (**F**) and mRNA expression (**G**) in isolated CAFs and tumor cells (Student's t test, n = 3). rhPOSTN was used as a positive control. Data are shown as means ± SEM. **, *P* < 0.01.

Next, we investigated the cellular source of POSTN in papillary thyroid tumors. As an extracellular protein, POSTN is mainly secreted by stromal cells in solid tumors 12, 14, 15. Immunofluorescence co-staining assays showed that POSTN mainly colocalized with α-SMA (CAF's marker) rather than with EpCAM (epithelial-derived cell's marker), CD31 (endothelial cell's marker) or F4/80 (macrophage's marker) in mouse xenograft tumors (**Figure 2A-B**) and clinical papillary thyroid tumor samples (**Figure S1A-B**). Immunohistochemical staining of mouse xenograft tumors (**Figure 2C**) and clinical papillary thyroid tumors (**Figure S1C**) also revealed that POSTN was deposited in CAFs but not in tumor cells. To further analyze the spatial distribution of *Postn* in papillary thyroid tumor tissues, we performed fluorescence* in situ* hybridization and observed colocalization of *Postn* and *Acta2* transcripts in CAFs in mouse xenograft tumors (**Figure 2D**), and colocalization of *POSTN* and *ACTA2* in CAFs in human papillary thyroid tumors (**Figure S1D**). We also found that POSTN was not expressed in normal thyroid epithelial cell line (Nthy-ori-3-1) and papillary thyroid cancer cell lines (**Figure 2E**). To determine whether CAFs produce POSTN, CAFs and tumor cells were both isolated from mouse xenograft tumors. Isolated mouse CAFs exhibited higher levels of POSTN protein (**Figure 2F**) and mRNA (**Figure 2G**) compared to isolated tumor cells. Collectively, these results suggest that POSTN is mainly derived from CAFs in mouse and human papillary thyroid tumors.

### POSTN promotes papillary thyroid tumor growth *in vivo* and cell proliferation *in vitro*

**Figure 3:**
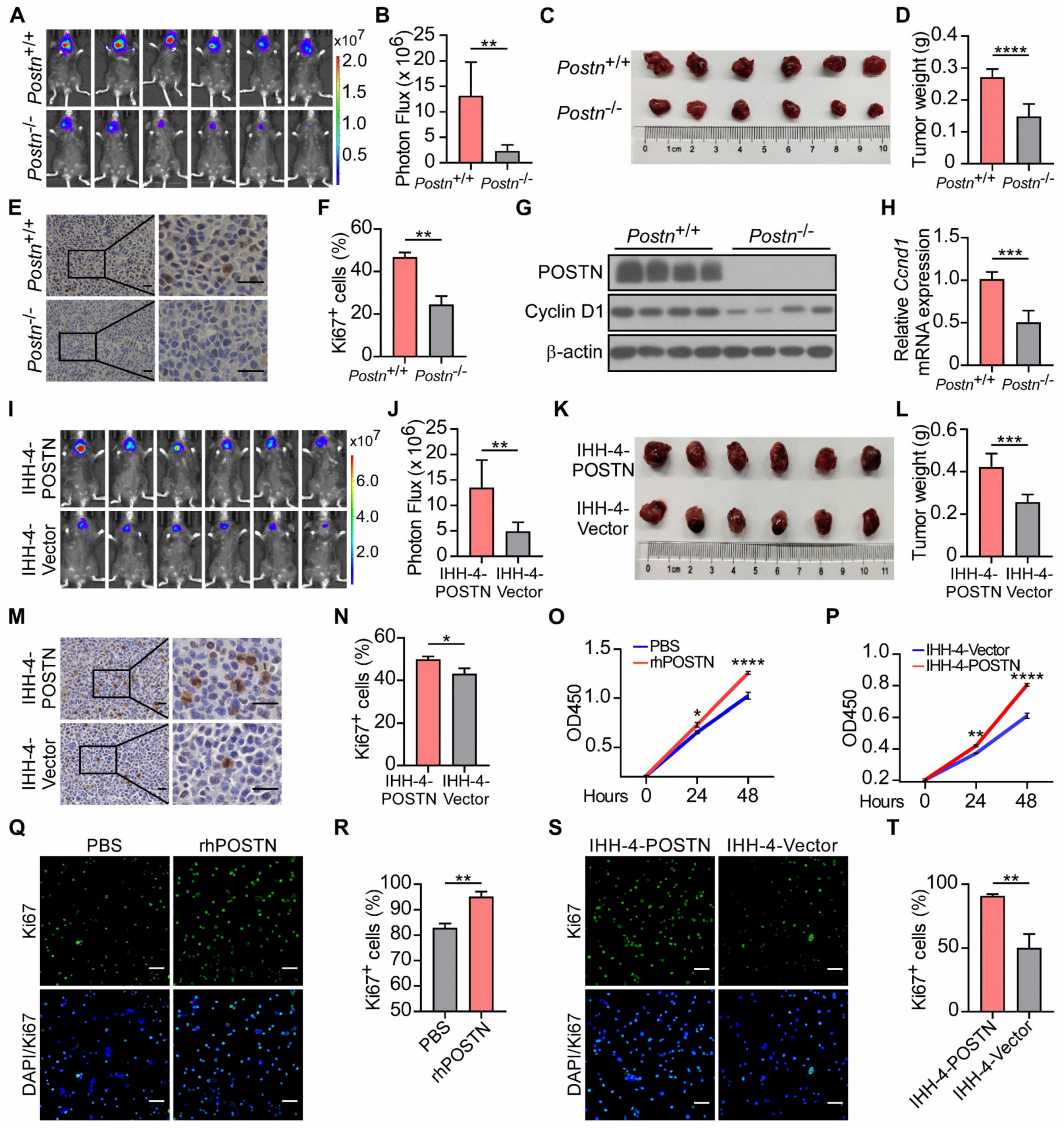
** POSTN deficiency inhibits tumor growth *in vivo*, whereas POSTN promotes IHH-4 cell growth *in vivo* and *in vitro*.** (**A** and **B**) Bioluminescence imaging (**A**) and quantification of tumor burden (**B**) in *Postn*^+/+^*Rag1*^-/-^ (*Postn*^+/+^) and *Postn*^-/-^*Rag1*^-/-^ (*Postn*^-/-^) mice after orthotopic injection with IHH-4 cells (n = 6). (**C** and **D**) Images (**C**) and quantification of weights (**D**) of isolated orthotopic tumors from mice in each group in (**A**) (n = 6). (**E** and **F**) Immunohistochemical staining for Ki67 (**E**) and quantitative analysis of Ki67^+^ cells (**F**) in tumor tissues. Scale bars, 25 μm (n = 3). (**G**) Western blot analysis of POSTN and Cyclin D1 in orthotopic tumor tissues from *Postn*^+/+^*Rag1*^-/-^ and *Postn*^-/-^*Rag1*^-/-^ mice. (**H**) qRT-PCR analysis of *Cyclin D1* (*Ccnd1*) in orthotopic tumor tissues from *Postn*^+/+^*Rag1*^-/-^ and *Postn*^-/-^*Rag1*^-/-^ mice (n = 4). (**I** and **J**) Bioluminescence imaging (**I**) and quantification of tumor burden (**J**) in *Postn*^-/-^*Rag1*^-/-^ mice after orthotopic injection with IHH-4 cells transfected with POSTN or control vector (n = 6). (**K** and **L**) Images (**K**) and quantification of weights (**L**) of isolated orthotopic tumors from mice in each group in (**I**) (n = 6). (**M** and **N**) Immunohistochemical staining for Ki67 (**M**) and quantitative analysis of Ki67^+^ cells (**N**) in tumor tissues. Scale bars, 25 μm (n = 3). (**O**) Analysis of the proliferation of IHH-4 cells treated with or without 100 ng/mL rhPOSTN for the indicated time by CCK8 assay (n = 3). (**P**) Analysis of the proliferation of IHH-4 cells transfected with POSTN or control vector (n = 3). (**Q** and **R**) Representative images of the immunofluorescence staining of Ki67 (**Q**) and quantification of Ki67^+^ cells (**R**) in IHH-4 cells treated with or without 100 ng/mL rhPOSTN. Scale bars, 50 μm (n = 3). (**S** and **T**) Representative images of the immunofluorescence staining of Ki67 (**S**) and quantification of Ki67^+^ cells (**T**) in IHH-4 cells transfected with POSTN or control vector. Scale bars, 50 μm (n = 3). Data are shown as means ± SEM. Student's t test (B, D, F, H, J, L, N, R and T). Two-way ANOVA (O and P). *, *P* < 0.05; **, *P* < 0.01; ***, *P* < 0.001; ****, *P* < 0.0001.

We further employed* Postn*^-/-^*Rag1*^-/-^ mice to assess the influence of POSTN deficiency on papillary thyroid tumor formation. Two weeks after orthotopic injection, tumor growth was monitored by bioluminescence imaging. *Postn*^-/-^*Rag1*^-/-^ mice exhibited significantly reduced tumor growth and decreased tumor weight compared to *Postn*^+/+^*Rag1*^-/-^ mice (**Figure 3A-D**, **Figure S2A-D**). In addition, mouse xenograft tumor tissues from *Postn*^-/-^*Rag1*^-/-^ mice displayed lower cell proliferation compared to tumors from *Postn*^+/+^*Rag1*^-/-^ mice (**Figure 3E-F**, **Figure S2E-F**). The protein and mRNA levels of cell proliferation marker Cyclin D1 were lower in the tumor tissues of *Postn*^-/-^*Rag1*^-/-^ mice than those in *Postn*^+/+^*Rag1*^-/-^ mice (**Figure 3G-H**). These data demonstrate that POSTN deficiency inhibits tumor growth of papillary thyroid tumors *in vivo*.

To interrogate the impact of endogenous POSTN on papillary thyroid tumor propagation, we ectopically expressed POSTN in IHH-4 and TPC-1 cells (**Figure S2G-H**). These cell lines were also stably labeled with a luciferase reporter and orthotopically injected into thyroid glands to generate xenograft tumors. Tumor cells transfected with POSTN vector (POSTN-OE) showed significantly accelerated tumor growth compared to the control group (**Figure 3I-L**, **Figure S2I-L**). POSTN-OE orthotopic tumors had more Ki67^+^ cells than the control group (**Figure 3M-N**, **Figure S2M-N**). Cell proliferation assay and immunofluorescence staining also showed that recombinant human POSTN protein (rhPOSTN) treated IHH-4 and TPC-1 cells displayed enhanced proliferative capability compared to control cells (**Figure 3O**,** Q**-**R**, **Figure S2O**,** Q**-**R**). Moreover, POSTN-OE tumor cells exhibited increased proliferative potential in comparison to control cells (**Figure 3P**,** S-T**, **Figure S2P**,** S-T**). These findings indicate that POSTN promotes papillary thyroid tumor growth *in vivo* and tumor cell proliferation *in vitro*.

### Deficiency of POSTN in CAFs impairs CAF-promoted papillary thyroid tumor growth *in vivo* and cell proliferation *in vitro*

**Figure 4:**
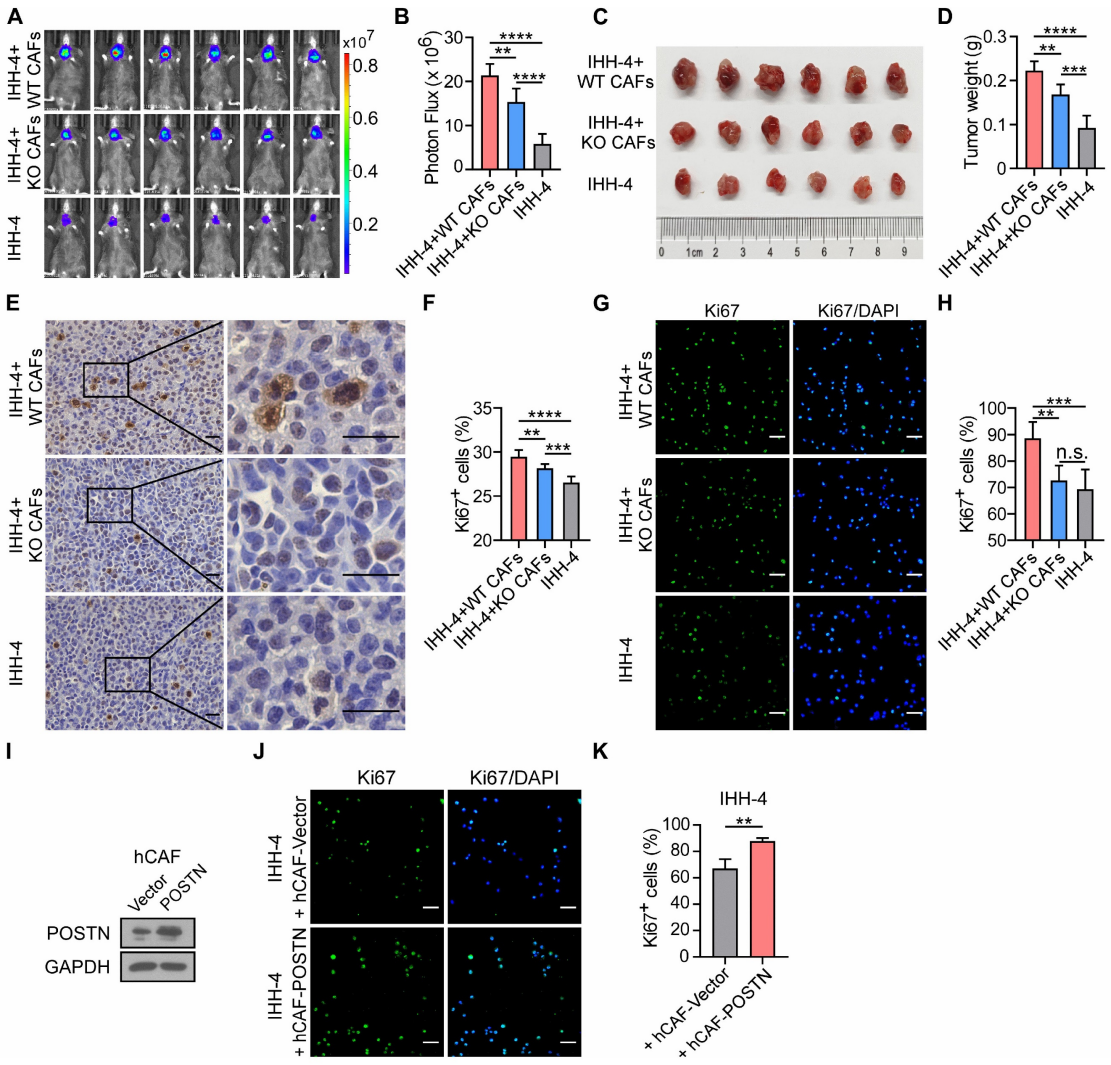
** Deficiency of POSTN in CAFs impairs CAF-promoted papillary thyroid tumor growth *in vivo* and IHH-4 cell proliferation *in vitro.*** (**A** and **B**) Bioluminescence imaging (**A**) and quantification of tumor burden (**B**) of* Postn*^-/-^*Rag1*^-/-^ mice after orthotopic injection with IHH-4 cells alone or together with WT or KO CAFs (n = 6). (**C** and **D**) Images (**C**) and quantification of weights (**D**) of isolated orthotopic tumors from mice in each group in (**A**) (n = 6). (**E** and **F**) Immunohistochemical staining for Ki67 (**E**) and quantitation of Ki67^+^ cells (**F**) in tumor tissues from each group in (**A**). Scale bars, 25 μm (n = 6). (**G** and **H**) Immunofluorescence images (**G**) and quantitation (**H**) of Ki67^+^ IHH-4 cells cultured alone or co-cultured with WT or KO CAFs (n = 6). (**I**) Western blot analysis of POSTN in human CAFs transfected with POSTN or control vector. (**J** and **K**) Immunofluorescence images (**J**) and quantitation (**K**) of Ki67^+^ IHH-4 cells co-cultured with the vector control or POSTN-overexpressing human CAFs (n = 4). Data are shown as means ± SEM. One-way ANOVA (B, D, F and H). Student's t test (K). **, *P* < 0.01; ***, *P* < 0.001; ****, *P* < 0.0001; n.s., no significant difference.

To further address the role of CAF-derived POSTN in papillary thyroid tumor growth, CAFs were isolated from *Postn*^+/+^*Rag1*^-/-^ (WT CAFs) or *Postn*^-/-^*Rag1*^-/-^ (KO CAFs) mouse orthotopic thyroid tumors and we orthotopically co-injected tumor cells alone or together with WT CAFs or KO CAFs into *Postn*^-/-^*Rag1*^-/-^ mice. Two weeks after implantation, tumor burden of mice was evaluated by bioluminescence imaging. A significant increase of tumor burden and weight was found in mice co-injected with tumor cells and WT CAFs compared with tumor cells alone, whereas POSTN depletion in co-injected CAFs significantly impaired the CAF-promoted growth of papillary thyroid tumor (**Figure 4A-D**, **Figure S3A-D**). Moreover, immunohistochemical staining further revealed that orthotopic tumors from mice co-injected with tumor cells and WT CAFs exhibited significant upregulation of Ki67^+^ cells compared to tumors from mice injected with tumor cells alone or together with KO CAFs (**Figure 4E-F**, **Figure S3E-F**).

To further investigate the effect of CAFs with or without POSTN on papillary thyroid tumor cell growth, we cultured tumor cells alone or co-cultured with WT CAFs or KO CAFs. Cocultivation of tumor cells with WT CAFs increased the proportion of Ki67^+^ tumor cells, compared to cocultivation with KO CAFs or without CAFs (**Figure 4G-H**; **Figure S3G-H**). To directly assess the impact of human CAF (hCAF)-derived POSTN on papillary thyroid tumor growth, hCAFs were isolated from human papillary thyroid tumor samples and stably overexpressed POSTN (hCAF-POSTN) (**Figure 4I**). Cocultivation of tumor cell lines with hCAF-POSTN increased the proportion of Ki67^+^ tumor cells, compared to cocultivation with hCAF-Vector (**Figure 4J-K**; **Figure S3I-J**). Taken together, these results suggest that CAF-derived POSTN promotes papillary thyroid tumor growth and deficiency of POSTN in CAFs impairs CAF-promoted papillary thyroid tumor growth *in vivo* and cell proliferation *in vitro*.

### POSTN promotes cell proliferation and the expression of IL-4 in papillary thyroid tumor cells by activating the integrin-FAK-STAT3 signaling

**Figure 5:**
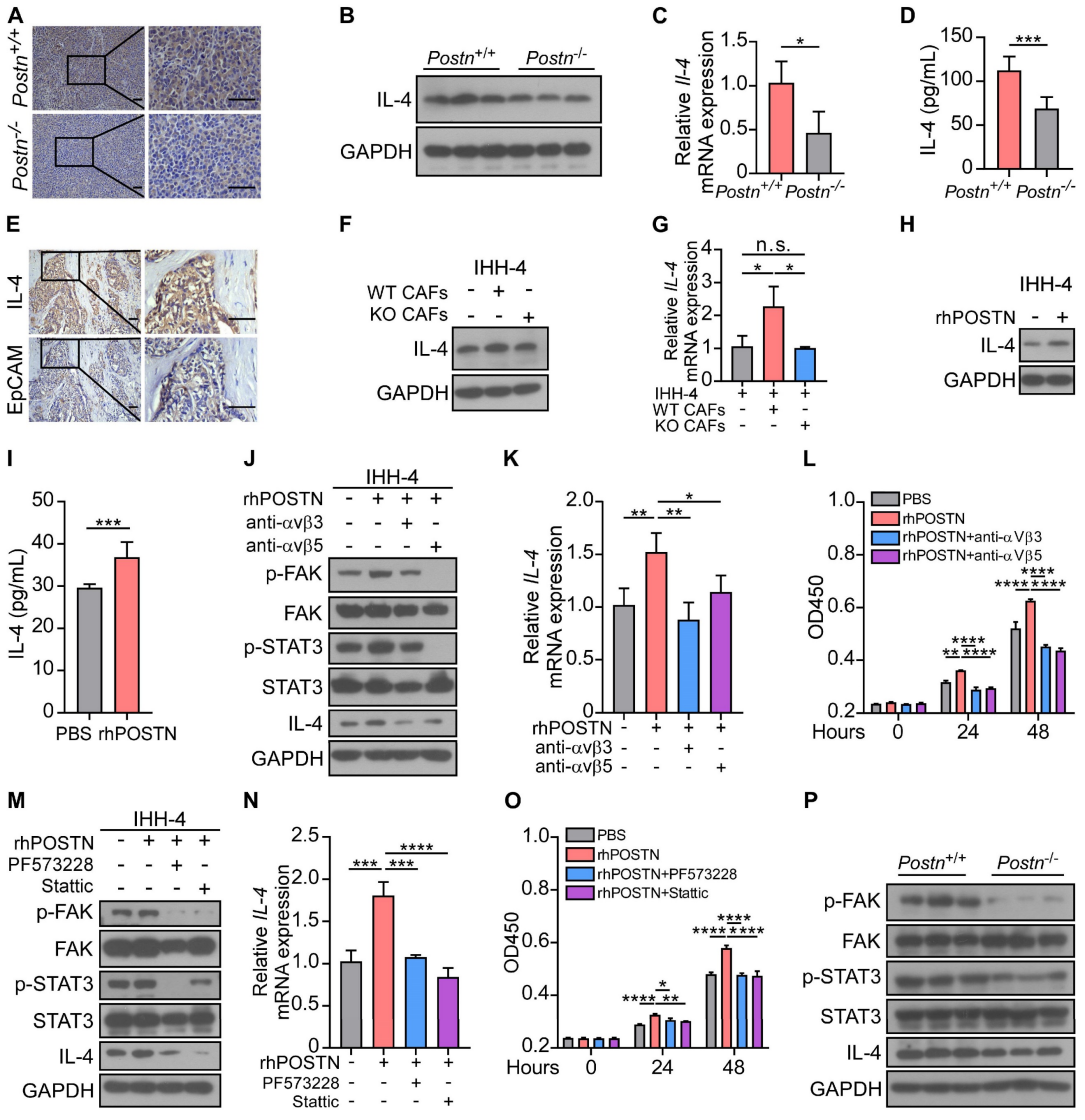
** POSTN promotes the proliferation and IL-4 expression in IHH-4 cells by activating the integrin-FAK-STAT3 signaling.** (**A**) Immunohistochemical analysis of the expression of IL-4 in tumor tissues from *Postn*^+/+^*Rag1*^-/-^ and *Postn*^-/-^*Rag1*^-/-^ mice. Scale bars, 50 μm. (**B**-**D**) Western blot (**B**), qRT-PCR (**C**) (n = 4) and ELISA (**D**) (n = 6) analyses of IL-4 expression in tumor tissues of *Postn*^+/+^*Rag1*^-/-^ and *Postn*^-/-^*Rag1*^-/-^ mice. (**E**) Immunohistochemical analysis of the expression of IL-4 and EpCAM in human papillary thyroid tumor. Scale bars, 50 μm. (**F** and **G**) Western blot (**F**) and qRT-PCR (**G**) analyses of IL-4 expression in IHH-4 cells cultured alone or co-cultured indirectly with WT or KO CAFs (n = 3). (**H** and **I**) Western blot (**H**) and ELISA (**I**) analyses of IL-4 level in IHH-4 cells treated with or without 100 ng/mL rhPOSTN (n = 6). (**J** and **K**) Western blot (**J**) and qRT-PCR (**K**) analyses of the indicated proteins and genes in IHH-4 cells treated with 100 ng/mL rhPOSTN in combination with antibodies against integrins αvβ3 or αvβ5 (n = 4). (**L**) Analysis of the proliferation of IHH-4 cells treated as in (**J**) for the indicated time by CCK8 assay (n = 4). (**M** and **N**) Western blot (**M**) and qRT-PCR (**N**) analyses of the indicated proteins and genes in IHH-4 cells treated with 100 ng/mL rhPOSTN alone or together with the indicated inhibitors (n = 3). (**O**) Analysis of the proliferation of IHH-4 cells treated as in (**M**) for the indicated time by CCK8 assay (n = 3). (**P**)Western blot analysis of the indicated proteins in the tumor tissues of *Postn*^+/+^*Rag1*^-/-^ and *Postn*^-/-^*Rag1*^-/-^ mice with papillary thyroid tumors. Data are shown as means ± SEM. Student's t test (C, D and I). One-way ANOVA (G, K and N). Two-way ANOVA (L and O). *, *P* < 0.05; **, *P* < 0.01; ***, *P* < 0.001; ****, *P* < 0.0001; n.s., no significant difference.

Our above investigation showed that co-cultured with CAFs enhanced tumor cell proliferation. This intriguing connection led us to explore the potential cytokines from tumor cells that might be responsible for driving CAF-derived POSTN-induced progression of papillary thyroid tumors. TGFβ, IL-4, IL-13, IL-6, CCL2, FGF1 and PDGF have been demonstrated to promote the production of POSTN by stromal cells 11, 12, 16, 17. We found that *IL-4* was significantly upregulated in papillary thyroid tumor cells treated with rhPOSTN (**Figure S4A**). Previous studies revealed that thyroid tumor cells in which IL-4 is produced via autocrine, thus promoting tumor progression and resistance to chemotherapy drugs 18, 19. However, the molecular mechanism underlying the role of IL-4 in thyroid tumors remains elusive. Immunohistochemical staining, western blot, qRT-PCR and ELISA analyses further confirmed that IL-4 was significantly down-regulated in the tumor tissues of *Postn*^-/-^*Rag1*^-/-^ injected with IHH-4 cells compared to their *Postn*^+/+^*Rag1*^-/-^ counterparts (**Figure 5A-D**). Immunohistochemical staining and western blot analyses revealed that IL-4 was mainly produced by epithelial tumor cells rather than CAFs (**Figure 5E**, **Figure S4B**). We observed a notable increase in both protein and mRNA levels of IL-4 in tumor cells indirectly co-cultured with WT CAFs compared to those cultured alone or co-cultured with KO CAFs (**Figure 5F-G**, **Figure S4C-D**). Moreover, rhPOSTN-treated tumor cells had a higher IL-4 level than PBS-treated cells (**Figure 5H-I**, **Figure S4E-G**).

Previous studies revealed that POSTN serves as a ligand of its receptors integrins (such as αvβ3 and αvβ5), facilitating cell proliferation and migration 12, 20. However, the underlying mechanisms of CAF-derived POSTN in the progression of papillary thyroid tumors remain unclear. Thus, we examined the downstream signaling of POSTN and found that rhPOSTN treatment increased the levels of phosphorylated FAK (p-FAK), phosphorylated STAT3 (p-STAT3) and IL-4 in papillary thyroid tumor cells, whereas showing no obvious effect on phosphorylated ERK (p-ERK) or phosphorylated Src (p-Src). Integrin neutralizing antibodies αvβ3 and αvβ5 blocked the upregulation of p-FAK, p-STAT3 and IL-4 in papillary thyroid tumor cells (**Figure 5J-K**, **Figure S4H-J**). Cell proliferation assay showed similar results that integrin neutralizing antibodies αvβ3 and αvβ5 reversed the elevated proliferative ability of tumor cells induced by POSTN (**Figure 5L**, **Figure S4K**). Furthermore, western blot and qRT-PCR assays revealed that FAK inhibitor PF573228 efficiently down-regulated p-STAT3 and IL-4 levels and the STAT3 inhibitor Stattic inhibited the POSTN-induced IL-4 upregulation in tumor cells (**Figure 5M-N**, **Figure S4L-M**). Cell proliferation assay confirmed that FAK inhibitor PF573228 and STAT3 inhibitor Stattic reversed the elevated proliferative ability of papillary thyroid tumor cells induced by POSTN (**Figure 5O**, **Figure S4N**). In the orthotopic mouse model, p-FAK, p-STAT3 and IL-4 were significantly decreased in tumor tissues of *Postn*^-/-^*Rag1*^-/-^ mice compared to *Postn*^+/+^*Rag1*^-/-^ mice (**Figure 5P**). Collectively, these data suggest that POSTN promotes proliferation and IL-4 production in tumor cells through the integrin-FAK-STAT3 signaling.

### IL-4-STAT6 signaling is involved in CAF activation and POSTN expression

**Figure 6:**
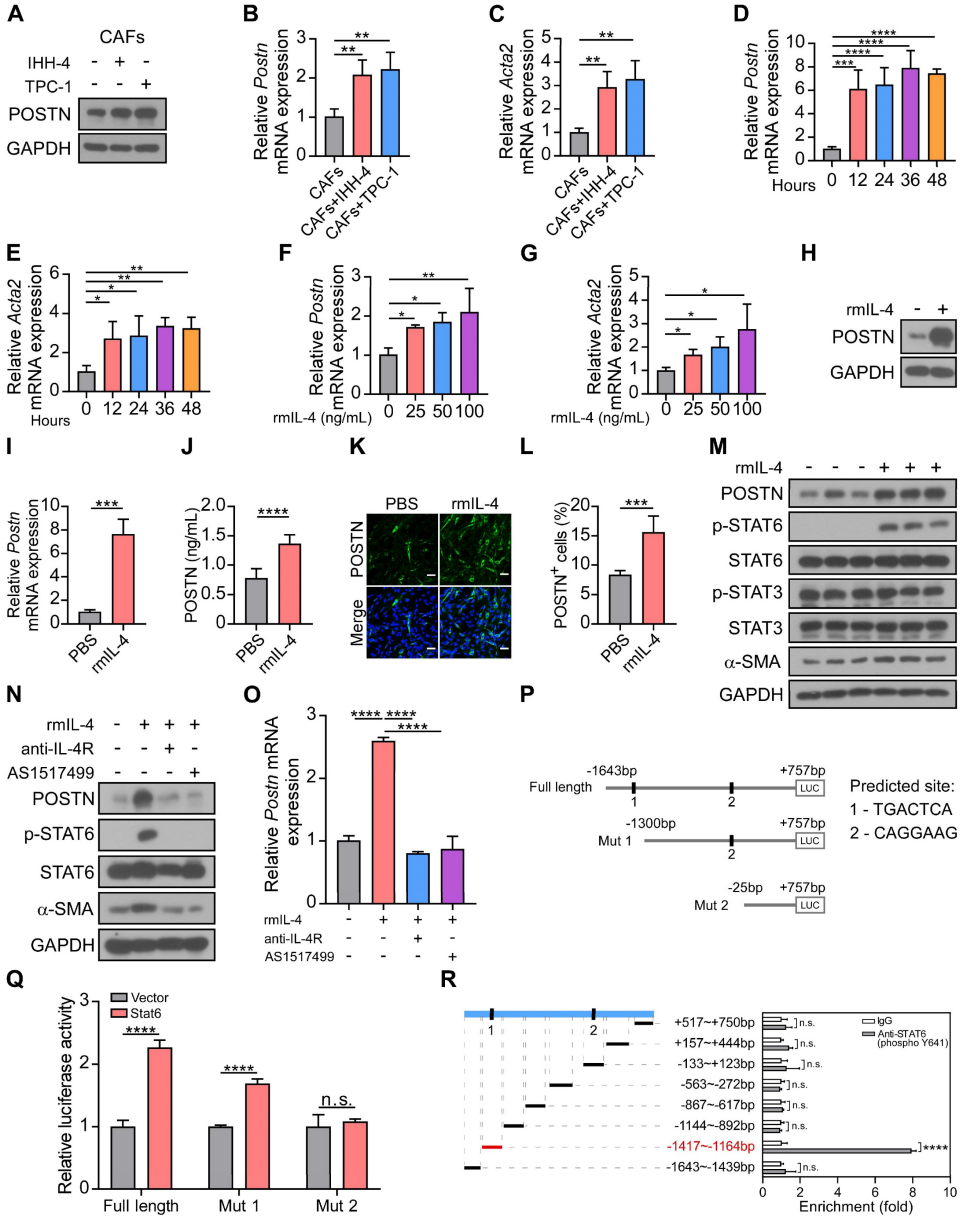
** IL-4 enhances the activation and POSTN expression in CAFs.** (**A**-**C**) Western blot (**A**) and qRT-PCR (**B** and **C**) analyses of POSTN and Acta2 in CAFs cultured alone or co-cultured indirectly with IHH-4 or TPC-1 cells (n = 4). (**D** and **E**) Relative mRNA levels of *Postn* (**D**) and *Acta2* (**E**) in isolated CAFs treated with rmIL-4 for the indicated time (n = 4). (**F** and **G**) Relative mRNA levels of *Postn* (**F**) and *Acta2* (**G**) in isolated CAFs treated with rmIL-4 for the indicated doses for 24 hours (n = 3). (**H** and **I**) Western blot (**H**) and qRT-PCR (**I**) analyses of POSTN in CAFs treated with rmIL-4 (n = 3). (**J**) ELISA analysis of POSTN level in CAFs treated with rmIL-4 (n = 6). (**K** and **L**) Immunofluorescence images (**K**) and quantitation (**L**) of POSTN^+^ cells in CAFs treated with or without rmIL-4. Scale bars, 50 μm (n = 6). (**M**-**O**) Western blot (**M** and **N**) and qRT-PCR (**O**) analyses of indicated proteins and *Postn* gene in CAFs treated with rmIL-4 alone or together with indicated inhibitors (n = 3). (**P**) Schematic representation of full length and two truncated luciferase reporters of mouse* Postn* promoter, and predicted binding sequences were predicted through a website-based search (https://guolab.wchscu.cn/hTFtarget/). (**Q**) Relative luciferase assay showing the mouse *Postn* promoter activity after co-transfected with vector Stat6 or vector control in HEK 293T cells (n = 3). (**R**) Relative enrichment of Stat6 on the indicated regions of *Postn* promoter in IL-4-treated CAFs detected by ChIP-qPCR assay using anti-STAT6 (phospho Y641) or control IgG antibody (n = 3). Data are shown as means ± SEM. One-way ANOVA (B-G and O). Student's t test (I, J and L). Two-way ANOVA (Q and R). *, *P* < 0.05; **, *P* < 0.01; ***, *P* < 0.001; ****, *P* < 0.0001; n.s., no significant difference.

IL-4 and IL-13 have been demonstrated to enhance POSTN expression and secretion in subepithelial fibrosis in bronchial asthma 21. We proposed that papillary thyroid tumor cell-derived IL-4 may in turn induce CAF activation and secretion of POSTN. Western blot and qRT-PCR analyses showed that POSTN and CAF activation marker *Acta2* expression were up-regulated in CAFs co-cultured with IHH-4 or TPC-1 tumor cells compared with CAFs cultured alone (**Figure 6A-C**). To investigate the role of IL-4, we examined the effect of recombinant mouse IL-4 protein (rmIL-4) treatment on isolated CAFs. CAFs were activated by IL-4 stimulation and relative mRNA levels of *Postn* and *Acta2* were significantly increased during 12-48 hours (**Figure 6D-E**) and exhibited dose-dependent upregulation (**Figure 6F-G**). Furthermore, western blot, qRT-PCR and ELISA analyses collectively affirmed that IL-4 stimulation significantly elevated POSTN levels in CAFs (**Figure 6H-J**). Immunofluorescence staining also confirmed that IL-4 stimulated CAFs to produce more POSTN (**Figure 6K-L**, **Figure S5A-B**).

Previous work indicated that IL-4 binds to its receptors IL-4R and subsequently activates STAT6 signaling 22, 23. We detected the downstream of IL-4 and found that α-SMA (CAF activation marker) and phosphorylated STAT6 (p-STAT6), rather than p-STAT3 were significantly up-regulated in CAFs treated with rmIL-4 protein (**Figure 6M**). Anti-IL-4R antibody and STAT6 inhibitor AS1517499 efficiently blocked IL-4-induced CAF activation and upregulation of POSTN in CAFs (**Figure 6N-O**). To further confirm whether transcription factor Stat6 regulates *Postn* expression, two potential binding sites of Stat6 were predicted in the region of mouse *Postn* promoter (-1643 bp to +757 bp) through the hTFtarget database. The luciferase reporter of *Postn* promoter and two truncations (Mut 1, Mut 2) were successfully constructed (**Figure 6P**). As shown in Figure 6Q, the dual-luciferase reporter assay demonstrated that Stat6 markedly increased the relative luciferase activity of the *Postn* promoter compared to the empty vector. Two truncations showed reduced luciferase activity in HEK 293T cells. To further verify the regulatory role of Stat6 on the *Postn* promoter, we performed the chromatin immunoprecipitation (ChIP) assay. ChIP-qPCR analysis confirmed that -1417 bp to -1164 bp of the *Postn* promoter might be the main binding region for Stat6 (**Figure 6R**). Our results reveal that Stat6 as a transcription factor regulates POSTN expression. Taken together, IL-4-STAT6 signaling is involved in CAF activation and POSTN expression.

### POSTN positively correlates with IL-4 expression in clinical papillary thyroid tumors

**Figure 7:**
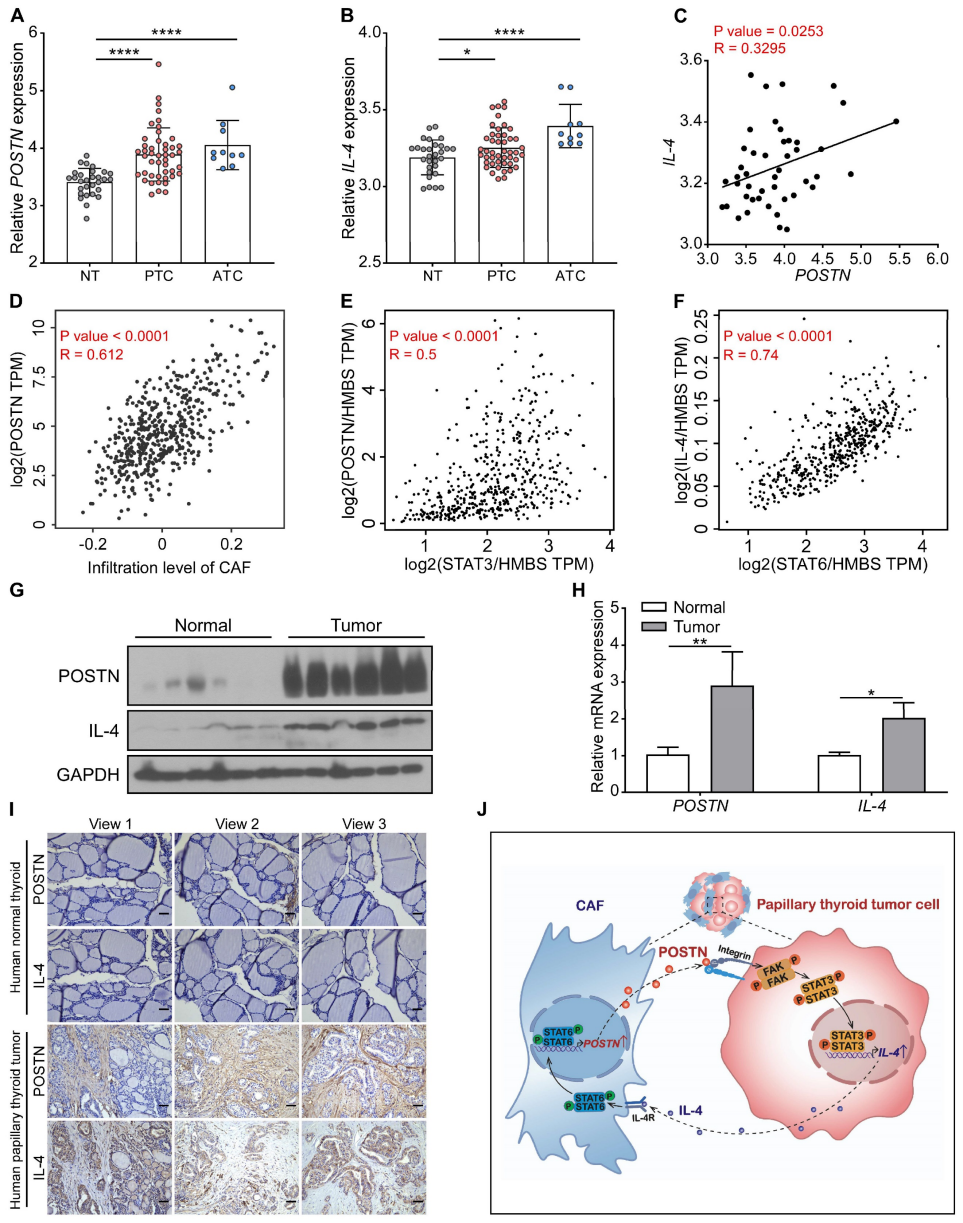
** The correlation between POSTN and IL-4 expression in human thyroid tumors.** (**A** and **B**) The expression of POSTN and IL-4 in normal thyroid tissues (NT, n = 29), papillary thyroid cancer [PTC, n = 46] and anaplastic thyroid cancer [ATC, n = 10] was analyzed in the GSE33630 dataset. One-way ANOVA with multiple comparisons test. (**C**) Spearman correlation analysis of IL-4 with POSTN of human papillary thyroid cancer in the GSE33630 database (n = 46). Spearman correlation coefficient (R). (**D**) Spearman correlation analysis of POSTN expression with infiltration level of CAF of human thyroid cancer in the Timer 2.0 database. (**E**) Spearman correlation analysis of POSTN with STAT3 of human thyroid cancer in the GEPIA database. Normalized by HMBS. (**F**) Spearman correlation analysis of IL-4 with STAT6 of human thyroid cancer in the GEPIA database. Normalized by HMBS. (**G** and **H**) Western blot (**G**) and qRT-PCR (**H**) analyses of POSTN and IL-4 in clinical papillary thyroid tumors (n = 3). Two-way ANOVA with multiple comparisons test. (**I**) Representative immunohistochemical images of POSTN and IL-4 in clinical papillary thyroid tumors and their corresponding adjacent tissues. Scale bars, 50 μm. (**J**) Model indicating CAF-derived POSTN promotes papillary thyroid tumor cells to secrete IL-4 through the integrin-FAK-STAT3 signaling, which in turn IL-4 stimulates CAFs to produce POSTN via STAT6 activation. Data are shown as means ± SEM. *, *P* < 0.05; **, *P* < 0.01; ****, *P* < 0.0001.

We further analyzed the relationship between POSTN and IL-4 expression in papillary thyroid tumors. By analyzing 85 samples from the GSE33630 dataset, we found that POSTN and IL-4 were significantly up-regulated both in papillary thyroid cancer and anaplastic thyroid cancer tissues relative to normal thyroid tissues and POSTN levels were positively correlated with IL-4 in papillary thyroid cancer (**Figure 7A**-**C**). Timer 2.0 database analysis showed that the expression level of POSTN was positively correlated with CAF infiltration in thyroid cancer (**Figure 7D**). By analyzing microarray data from the GEPIA database, we also found that POSTN levels were positively correlated with STAT3 (**Figure 7E**) and IL-4 is strongly linked to STAT6 (**Figure 7F**) in thyroid cancer. Western blot, qRT-PCR and immunohistochemical staining assays demonstrated that the levels of POSTN and IL-4 were up-regulated in clinical papillary thyroid tumors compared to their matched adjacent normal tissues (**Figure 7G-I**). Multiplex immunofluorescence staining assays further revealed that IL-4 was well colocalization with cancer cell marker EpCAM and POSTN was mainly distributed in tumor stroma (**Figure S6A**). These data support that POSTN positively correlates with IL-4 expression in clinical papillary thyroid tumors.

## Discussion

Accumulating evidence indicates that stromal cell-derived POSTN can bind to tumor cell-surface receptors to upregulate integrin outside-in signaling to promote tumor cell proliferation in B cell acute lymphoblastic leukemia, colorectal, breast and liver cancers 11, 15, 24. Nevertheless, the role of POSTN in papillary thyroid cancer has not been fully explored. Here, we delved into the underlying mechanisms through which POSTN promotes papillary thyroid tumor growth. We revealed that CAF-derived POSTN promotes papillary thyroid tumor cell proliferation and IL-4 expression via the integrin-FAK-STAT3 signaling, which in turn IL-4 stimulates CAFs to produce POSTN via STAT6 activation (**Figure 7J**). We uncover a feedback loop involving tumor cell-derived IL-4 and CAF-derived POSTN, which highlights the intricate crosstalk between tumor cells and their microenvironment. It raises the intriguing possibility that inhibiting IL-4 and POSTN might serve as a strategy to suppress the progression of papillary thyroid tumors.

Tumor cells can secrete several pro-fibrotic growth factors and inflammatory factors to activate stromal cells and remodel tumor microenvironment 25. As a key element of the tumor microenvironment, CAFs can promote tumor growth and modulate therapy responses through production of growth factors, cytokines and exosomes 26-28. In bladder cancer, SLC14A1^+^ CAFs produce WNT5A to confer stemness to bladder tumor cells, in turn tumor cells drive interferon production to enhance SLC14A1^+^ CAF differentiation 29. In pancreatic ductal adenocarcinoma, tumor cells release TGF-β to activate CAFs and then activated CAFs regulate ECM remodeling to support tumor cell migration 30. High CAF scores as a represent risk factor, have been reported to be positively correlated with predicting tumor aggressiveness, lymph node metastasis and poor prognosis in thyroid tumor patients 31. Our previous studies demonstrated that stromal cell-derived POSTN promotes cell proliferation and the expression of CCL2, IL-6 and TGFβ in tumor cells, which in turn these tumor cell-derived cytokines can increase the expression of POSTN in stromal cells, thus promoting tumor progression 11, 12, 32. In this study, we revealed that POSTN is predominantly derived from CAFs in papillary thyroid tumors and POSTN deficiency in CAFs impairs CAF-promoted tumor growth. While, recombinant IL-4 protein treatment increases POSTN expression in isolated CAFs via STAT6 activation. Therefore, IL-4-STAT6 signaling contributes to POSTN expression in activated CAFs, whereas POSTN-integrin-FAK-STAT3 signaling promotes IL-4 expression in tumor cells during papillary thyroid tumor tumorigenesis.

Previous studies indicated that FAK and STAT3 were highly expressed in papillary thyroid cancer cells compared with normal thyroid cells 33, 34. Moreover, FAK expression is critical for thyroid tumor tumorigenesis and growth 35, and activation of STAT3 enhances tumor cell proliferation and prevents apoptosis in most cancers 36. In line with this, we observed integrin neutralizing antibody, FAK inhibitor and STAT3 inhibitor significantly decreased the POSTN-induced IL-4 upregulation and reversed the POSTN-induced cell proliferation in papillary thyroid tumor cells. We also found that the levels of p-FAK, p-STAT3, and IL-4 in the tumors of *Postn*^-/-^*Rag1*^-/-^ mice were significantly reduced compared to the tumors from *Postn*^+/+^*Rag1*^-/-^ mice. This suggests that activated FAK and STAT3 may play critical roles in promoting papillary thyroid tumor progression.

IL-4 is a secreted and pleiotropic cytokine. Many studies demonstrated that IL-4 as an autocrine growth factor enhances tumor cell survival, apoptosis resistance and growth in thyroid cancer, pancreatic cancer, colorectal cancer and prostate cancer 37-40. Colon cancer stem cells autocrine produce and utilize IL-4 which enhances antiapoptotic protein expression to protect themselves from apoptosis in colorectal cancer 40. In non-small cell lung cancer, dual blockade of IL-4 and PD-1 enhances antitumor immune responses, proinflammatory cytokines production and upregulation of CD8^+^ T cells infiltration 41. In our study, we found that IL-4 was mainly produced by papillary thyroid tumor cells and IL-4 induced CAF activation and POSTN expression. Moreover, p-STAT6 was found to be higher in thyroid tumor cells compared with normal thyrocytes indicating that the STAT6 pathway is constitutively activated in thyroid tumor cells 37. Our results demonstrated that IL-4 binding to its receptors induces activation of transcription factor STAT6 and STAT6 may directly bind to *Postn* promoter to induce POSTN expression. Taken together, our study indicated that IL-4 and POSTN could serve as potential therapeutic targets to inhibit papillary thyroid tumor progression.

## Methods

### Animals

B6;129-*Postn^tm1Jmol^*/J (*Postn*^+/-^) mice were originally purchased from Jackson Laboratory, and underwent ten generations of breeding with wild-type C57BL/6J mice to establish the C57BL/6J *Postn*^+/-^ mice. B6.129S7-*Rag1^tm1Mom^*/J (*Rag1*^-/-^) mice were originally purchased from Jackson Laboratory. *Postn*^+/-^*Rag1*^+/-^ mice were generated by breeding *Postn*^+/-^*Rag1*^+/+^ and *Postn*^+/+^*Rag1*^-/-^ mice. Female *Postn*^+/-^*Rag1*^+/-^ and male *Postn*^+/-^*Rag1*^+/-^ mice were used to generate *Postn*^+/-^*Rag1*^-/-^ mice. *Postn*^+/+^*Rag1*^-/-^ and *Postn*^-/-^*Rag1*^-/-^ littermates for experiments were generated by crossing female* Postn*^+/-^*Rag1*^-/-^ and male *Postn*^+/-^*Rag1*^-/-^ mice. All mice were maintained under specific pathogen-free conditions at the Xiamen University Laboratory Animal Center. All experimental procedures involving mice were approved by the Animal Care and Use Committee of Xiamen University.

### Orthotopic mouse model of papillary thyroid tumor

The orthotopic model of papillary thyroid tumor was established by using 8- to 12-week-old *Postn*^+/+^*Rag1*^-/-^ and *Postn*^-/-^*Rag1*^-/-^ mice. In brief, 1 × 10^6^ tumor cells (IHH-4 or TPC-1) were suspended in 10 μL PBS and orthotopically injected into the right lobe of the thyroid gland 42. The mice were monitored two weeks later by bioluminescence imaging. To investigate the effect of POSTN deletion in CAFs on the proliferation of tumor cells in mice, 8- to 12-week-old *Postn*^-/-^*Rag1*^-/-^ mice were orthotopically injected with tumor cells alone (1 × 10^6^ cells, IHH-4 or TPC-1) or tumor cells plus *Postn*^+/+^ or *Postn*^-/-^ CAFs (1 × 10^5^ cells) and analyzed after two weeks. Mouse tumor samples were collected from orthotopic thyroid tumor-bearing mice and mouse normal thyroid tissues were collected from control mice for further western blot and qRT-PCR analyses.

### Patient samples

Fresh human papillary thyroid tumors and matched adjacent normal tissue samples (at least 2 cm from tumor margins) were collected under informed consent from the patients with greatest tumor diameter > 1 cm who underwent surgical resection and were without drug treatment previously. After excision, patient samples were fixed in 4% paraformaldehyde or stored at -80°C for further analysis. 6 paired papillary thyroid tumors and matched adjacent normal tissue samples were available for performing western blot and qRT-PCR analyses. All experiments on patient samples were performed in accordance with the approved guidelines of the Ethics Committees of the Zhongshan Hospital Affiliated to Xiamen University.

### Cell lines and culture

The HEK 293T cell line was purchased from the American Type Culture Collection (ATCC). Human papillary thyroid tumor cell line IHH-4 was purchased from JCRB Cell Bank. Human papillary thyroid tumor cell line TPC-1 was obtained from CLS Cell Lines Service GmbH. These cell lines were cultured in DMEM (Gibco) supplemented with 10% FBS (HyClone) and 1% penicillin and streptomycin (Gibco) and maintained at 37°C, 5% CO_2_ in a humidified incubator. All cell lines used for orthotopic mouse model experiments were stably transfected with a firefly luciferase expressing vector.

### Primary CAFs isolation

For CAF isolation, tumors were obtained from orthotopic thyroid tumors of *Postn*^+/+^*Rag1*^-/-^ or *Postn*^-/-^*Rag1*^-/-^ mice and human papillary thyroid tumor samples. CAFs were isolated from primary papillary thyroid tumor tissues by explant culture 43. Briefly, primary tumor tissues were finely sectioned and subjected to a 3-hour digestion at 37°C using a DMEM containing 2 mg/mL collagenase II and 20 μg/mL DNase I. The supernatant was filtered through 40 μm mesh strainer, followed by centrifugation. The pellet was resuspended in DMEM containing 10% FBS, 1% penicillin and streptomycin and seeded on a 3.5 cm dish. After 30 minutes incubation, non-adherent cells were washed away, leaving adhered CAFs. CAFs were used within 10 passages. The purity of the cultured CAFs was > 95%, validated by α-SMA staining.

### Cell co-culture assay

For the cell co-culture assay, CAFs were isolated from *Postn*^+/+^*Rag1*^-/-^ or *Postn*^-/-^*Rag1*^-/-^ orthotopic thyroid tumor-bearing mice. CAFs and tumor cells were co-cultured in two ways. The first method was seeding 5 × 10^5^ CAFs per well in 6-well plates and adding 5 × 10^4^ tumor cells to the 0.4 μm pore upper chamber (Corning, 3412) for 24 hours. The second method was seeding 5 × 10^5^ tumor cells in 6-well plates and adding 5 × 10^4^ CAFs to the 0.4 μm pore upper chamber for 24 hours.

### Immunohistochemical staining

Tissue samples fixed in 4% paraformaldehyde were embedded in paraffin and sliced into 4 μm sections. The slides were baked for 2 hours at 65°C and then deparaffinized. Antigen heat retrieval was performed with pH6.0 citric buffer or pH9.0 EDTA buffer. Tissue sections were then stained with primary antibodies detailed in Table S1. Tissues were mounted using UltraSensitive^TM^ SP (Mouse/Rabbit) IHC Kit (KIT-9710) and imaged with Leica DM4B upright microscope. Antibodies against POSTN (Adipoge, AG-20B-0033, 1:400), α-SMA (Cell Signaling Technology, 19245, 1:200), EpCAM (Abcam, ab213500, 1:200), Ki67 (Abcam, ab15580, 1:200) and IL-4 (Abcam, ab300138, 1:200) were used.

### Immunofluorescence staining

Tumor tissues were embedded in OCT (Sakura) by liquid nitrogen flash freezing. Tissue sections (4 μm thick) were baked at 37°C for 1 hour, fixed with ice acetone at -20°C for 20 minutes and washed with PBS. For cell immunofluorescence staining, cells on 20-mm diameter glass coverslips (NEST) were fixed with 4% paraformaldehyde at room temperature for 30 minutes and permeabilized with 0.4% Triton X-100 for 15 minutes. Slides or coverslips were blocked with 3% BSA and then incubated with the primary antibodies overnight at 4°C. After washing, slides or coverslips were incubated with secondary antibodies at room temperature for 1 hour and then incubated with DAPI for 30 minutes. Finally, slides or coverslips were washed with PBS, mounted and then imaged with confocal microscope (Zeiss LSM 780). The primary antibodies against: POSTN (Adipogen, AG-20B-0033, 1:400), α-SMA (Cell Signaling Technology, 19245, 1:200), EpCAM (Abcam, ab213500, 1:200), CD31 (Abcam, ab76533, 1:200), F4/80 (Abcam, ab300421, 1:200), Ki67 (Abcam, ab15580, 1:200) were used.

### Fluorescence *in situ* hybridization (FISH)

FISH was conducted using the Fluorescent *In Situ* Hybridization Kit (GenePharma, China) according to the manufacturer's instructions. The oligonucleotide modified probes labeled with FAM or TAMRA were designed and synthesized. Fresh paraffin-embedded tissue sections, 4 μm thick, were preheated at 65°C for 2 hours. After deparaffinization and rehydration, tissue sections were digested with Proteinase K solution for 20 minutes at 37°C and denatured for 8 minutes at 78°C. After dehydration, probes were mixed in the hybridization solution and incubated overnight at 37°C. Sections were washed and cell nuclei were counterstained with DAPI. Images were acquired using a confocal microscope. Sequences of designed FISH probes are listed in Table S2.

### Recombinant protein, antibody, and kinase inhibitor treatment

IHH-4 or TPC-1 cells were cultured with serum-free medium for 12 hours. Before treatment with 100 ng/mL rhPOSTN (R&D Systems, catalog no. 3548-F2-050), cells were incubated with anti-integrins αvβ3 (Merck/Millipore, MAB1876-Z, 2.5 μg/mL) or αvβ5 (Merck/Millipore, MAB1961, 2.5 μg/mL) antibodies and PF573228 (20 nM) (Selleck Chemicals, s2013), Stattic (1 μM) (Selleck Chemicals, s7024) for 30 minutes. Similarly, CAFs were cultured with serum-free medium for 6 hours, before treatment with 25 ng/mL rmIL-4 (Abclonal, RPO1161) or recombinant human IL-4 protein (rhIL-4) (Abclonal, RP00995), CAFs were incubated with anti-IL-4R antibody (Abcam, ab271041, 5 μg/mL) or AS1517499 (500 nM) (MedChemExpress, HY-100614) for 30 minutes. After 24 hours or indicated time, cells were collected and detected by qRT-PCR or western blot assays.

### Bioluminescence imaging

The tumors were monitored using bioluminescence imaging on day 14 after the construction of the orthotopic tumor model. Tumor-bearing mice were intraperitoneally injected with D-Luciferin Potassium Salt (Goldbio), and bioluminescence imaging signals were captured within 10 minutes after injection using small animal imaging technology (IVIS Lumina XRMS III).

### Luciferase reporter assay

HEK 293T cells were seeded in 24-well plates and co-transfected with pGL3-Postn-luciferase reporter or mutant constructs, Renilla and pCMV5-Stat6 or control plasmid. After 24 hours of transfection, the cells were lysed and luciferase activity was measured using the luminometer (Promega, E1910) and normalized to internal Renilla luciferase activity. The primers used for the construction of the plasmids are listed in Table S3.

### Cell proliferation assay

Cell proliferation assay was carried out using the Cell Counting Kit-8 (CCK8, Beyotime, C0038). Tumor cells were seeded in 96-well plates at a density of 2 × 10^3^ cells per well in 100 μL of medium. After 24 hours, cells were treated with antibody, kinase inhibitor or rhPOSTN protein. After treatments for the indicated time, 10 μL of CCK8 solution was added to each well. After 2 hours incubation, the absorbance of each well was determined at 450 nm with microplate photometer (multiskan FC).

### Chromatin immunoprecipitation (ChIP) assay

ChIP assay was performed using the ChIP Assay Kit (P2078; Beyotime Biotechnology) according to the manufacturer's instructions. Briefly, CAFs were cultured with serum-free medium for 6 hours, then treated with 25 ng/mL rmIL-4 for 24 hours. IL-4-treated CAFs were collected, followed by ChIP assays with anti-STAT6 phospho Y641 (Abcam, ab235591, 2 μg) or control IgG antibody (Sigma-Aldrich, I5006, 2 μg), the DNA fragments were used for quantitative PCR. The values are reported as fold enrichment of specific sequences within anti-STAT6 (phospho Y641) precipitated DNA relative to the anti-IgG control. The primers used for amplification the indicated regions of *Postn* promoter are listed in Table S4.

### Bioinformatic analysis

The differential analysis, stage-plot analysis, patient survival analysis and correlation analysis of gene expression in human thyroid cancer samples were analyzed via the website-based Gene Expression Profiling Interactive Analysis (GEPIA) database (http://gepia.cancer-pku.cn/) 44. Tumor Immune Estimation Resource (TIMER) database was used to evaluate the correlation between POSTN expression with CAFs infiltrated in human thyroid tumors (http://timer.cistrome.org) 45. The GSE33630 dataset was downloaded from the Gene Expression Omnibus database and differentially expressed genes were analyzed by the online analysis tool GEO2R (https://www.ncbi.nlm.nih.gov/geo/geo2r/). Then the correlation between the *POSTN* gene and *IL-4* gene was analyzed by Spearman correlation analysis.

### Statistical analysis

All data were shown as mean ± standard error of the mean (SEM). GraphPad Prism 8.0 software was used for statistical analysis, using statistical tests indicated in figure legends. All tests were performed three or more times to ensure reproducibility. For two-group comparisons, two-tailed Student's t test was used. For multiple groups comparisons, one-way ANOVA or two-way ANOVA with multiple comparisons test were performed. *P* value below 0.05 was considered statistically significant.

## Supplementary Material

Supplementary methods, figures and tables.

## Abbreviations

POSTN: periostin
CAFs: cancer-associated fibroblasts
TME: tumor microenvironment
ECM: extracellular matrix
FISH: fluorescence *in situ* hybridization
rhPOSTN: recombinant human POSTN protein
rmIL-4: recombinant mouse IL-4 protein
rhIL-4: recombinant human IL-4 protein
p-FAK: phosphorylated FAK
p-STAT3: phosphorylated STAT3
p-Src: phosphorylated Src
p-ERK: phosphorylated ERK
p-STAT6: phosphorylated STAT6
