# Supplementary material for: Cancer-associated fibroblast-derived periostin promotes papillary thyroid tumor growth through integrin-FAK-STAT3 signaling
Source: Theranostics. 2024 May 13;14(7):3014–28. doi: 10.7150/thno.94207 (PMC11103496; doi:10.7150/thno.94207)
Supplement: Supplementary file 1 — Supplementary methods, figures and tables. [file thnov14p3014s1.pdf]

Supplementary Material

Cancer-associated fibroblast-derived periostin promotes papillary thyroid tumor growth through integrin-FAK-STAT3 signaling

Xiaorui Jin,[1,2] Qianmei Deng,[1] Shuting Ye,[1] Shuting Liu,[1] Yilong Fu,[3] Yingfu Liu,[4] Guoyang Wu,[3*] Gaoliang Ouyang,[1,2*] and Tiantian Wu[1*]

Supplementary methods

Western blot

Total proteins were collected from tumor tissues or cells using RIPA lysis buffer, augmented with protease and phosphatase inhibitors (Roche). All samples were subjected to electrophoresis on 8%-14% SDS-PAGE gels and transferred onto PVDF membranes (Millipore). Membranes were blocked in 5% BSA at room temperature for 2 hours and incubated with primary antibodies at 4°C overnight. Subsequently, the membranes were washed with TBST for 30 minutes and incubated with HRP-conjugated secondary antibody at room temperature for 1 hour. After washing again, proteins were visualized through X-ray films. The primary antibodies used are listed in Table S1. The primary antibodies against: GAPDH (Cell Signaling Technology, 92310, 1:5000), POSTN (Adipogen, AG-20B-0033, 1:3000), Cyclin D1 (Cell Signaling Technology, 2978, 1:2000), β-actin (Cell Signaling Technology, 8457, 1:5000), IL-4 (Abcam, ab62351, 1:2000), p-FAK (Cell Signaling Technology, 3281, 1:2000), FAK (Abcam, ab40794, 1:2000), p-STAT3 (Cell Signaling Technology, 9145, 1:2000), STAT3 (Cell Signaling Technology, 9139, 1:2000), p-ERK1/2 (Cell Signaling Technology, 9101, 1:5000), ERK1/2 (Cell Signaling Technology, 9102, 1:5000), p-STAT6 (Abcam, ab188080, 1:2000) and STAT6 (Abclonal, A19120, 1:2000) were used.

qRT-PCR analysis

Total RNA was extracted from tumor cells or tissue samples using TriPure Isolation

Reagent (Roche) following the manufacturer's instructions. RNA was reverse transcribed into cDNA by using the ReverTra Ace qPCR RT kit (TOYOBO, FSQ-101) according to the manufacturer's instructions. qRT-PCR analysis was conducted on CFX Connect Real-Time PCR machine (Bio-Rad) using the BrightCycle Universal SYBR Green qPCR Mix with UDG (Abclonal, RK21219) in line with kit protocol. mRNA expression of target genes was normalized by the expression of *GAPDH*. Primer sequences are listed in the Table S5.

Generation of stable cell lines for POSTN overexpression

To generate stable cell lines for POSTN overexpression, the pCDH-CMV-POSTN plasmids were transfected along with lentiviral packaging plasmids (pCMV-VSVG, pRSV-REV and pMDL) into HEK 293T cells with polyethyleneimine to produce viruses. 48 hours after transfection, the viral supernatants were collected and filtered with 0.22 μm filters. Lentiviruses were concentrated by ultracentrifugation. Target cells were infected with the lentiviruses, and selected with puromycin to generate stable cell lines. After puromycin selection, the cell lines of POSTN overexpression were validated by western blot analysis.

ELISA assay

Cytokines in the orthotopic mouse tumor tissues and cells were detected with mouse POSTN ELISA kits (R&D systems, AF2955-SP) or human IL-4 ELISA kit (Abclonal, RK00003) according to the manufacturer's instructions.

Multiplex immunofluorescence staining

Multiplex immunofluorescence staining assay was performed on human papillary thyroid tumors to explore the distributions of POSTN and IL-4 expression using the TSA Fluorescence Triple Staining Kit (Abclonal, RK05903) according to the manufacturer's instructions. Antibodies against POSTN (Adipoge, AG-20B-0033, 1:400), EpCAM (Abcam, ab213500, 1:200) and IL-4 (ABclonal, A5649, 1:200) were used.

Supplementary figures

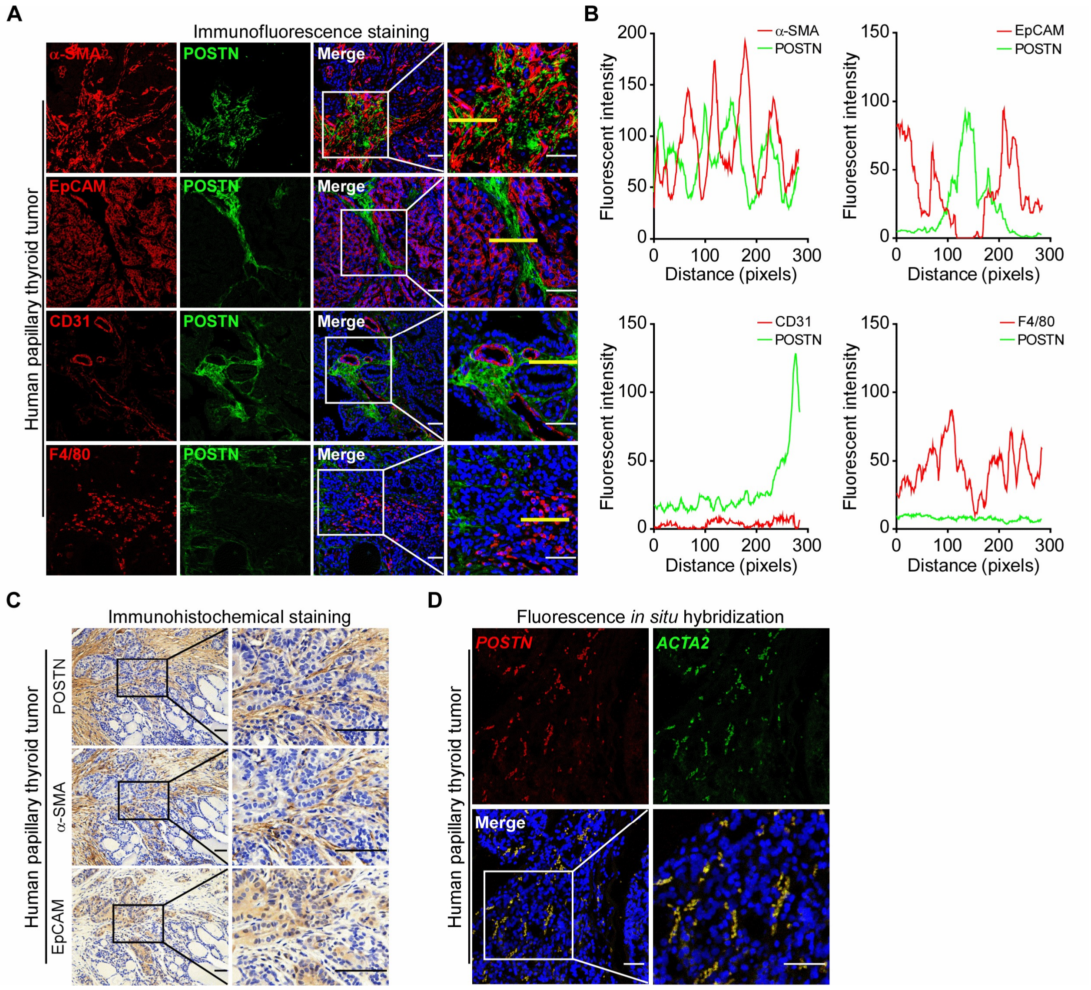

Figure S1. POSTN is mainly derived from CAFs in human papillary thyroid tumors. (**A**) Representative immunofluorescence co-staining of POSTN with α-SMA, EpCAM, CD31 or F4/80 in human papillary thyroid tumors. Nuclei were counterstained with DAPI. Scale bars, 50 μm. (**B**) Plots of fluorescent intensity along the yellow line of images in (**A**). (**C**) Representative immunohistochemical staining of POSTN, α-SMA and EpCAM in the human papillary thyroid tumors. Scale bars, 50 μm. (**D**) Fluorescence *in situ* hybridization of *POSTN* and *ACTA2* in human papillary thyroid tumors. Nuclei were counterstained with DAPI. Scale bars, 50 μm.

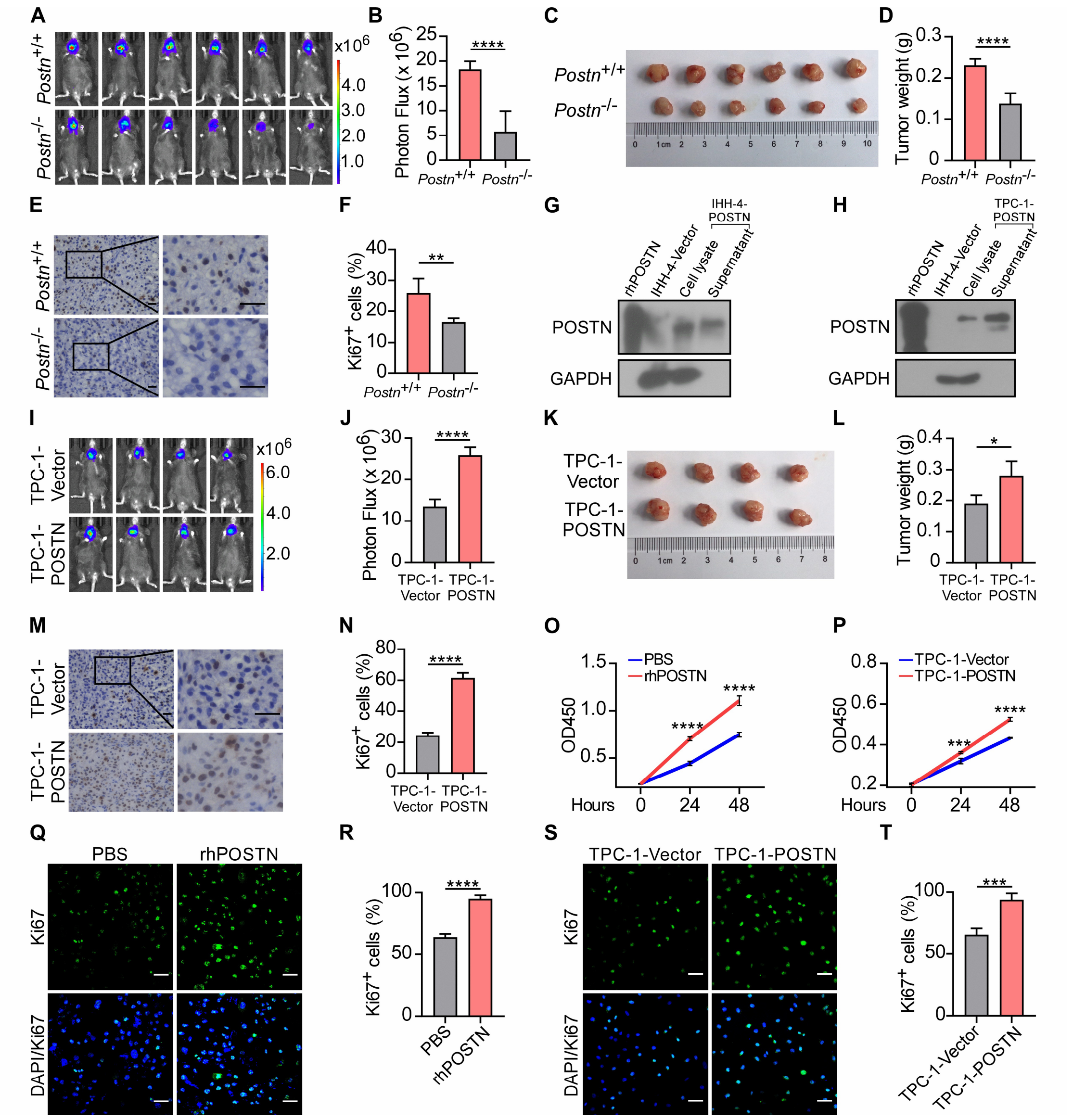

Figure S2. POSTN deficiency inhibits tumor growth *in vivo*, whereas POSTN promotes TPC-1 cell growth *in vivo* and *in vitro*. (**A** and **B**) Bioluminescence imaging (**A**) and quantification of tumor burden (**B**) in *Postn*$^{+/+}$*Rag1*$^{-/-}$ (*Postn*$^{+/+}$) and *Postn*$^{-/-}$*Rag1*$^{-/-}$ (*Postn*$^{-/-}$) mice after orthotopic injection with TPC-1 cells (n = 6). (**C** and **D**) Images (**C**) and quantification of weights (**D**) of isolated orthotopic tumors from mice in each group in (**A**) (n = 6). (**E** and **F**) Immunohistochemical staining for Ki67 (**E**) and quantitative analysis of Ki67^{+} cells (**F**) in tumor tissues from each group in (**A**). Scale bars, 25 μm (n = 4). (**G** and **H**) Western blot analysis of POSTN protein level in total cell lysates and culture supernatants of IHH-4 (**G**) or TPC-1 (**H**) cells with either vector or POSTN stably expressed. rhPOSTN was used as a positive control. (**I** and **J**) Bioluminescence imaging (**I**) and quantification of tumor burden (**J**)

in *Postn*$^{-/-}$*Rag1*$^{-/-}$ mice after orthotopic injection with TPC-1 cells transfected with POSTN or control vector (n = 4). (**K** and **L**) Images (**K**) and quantification of weights (**L**) of isolated orthotopic tumors from mice in each group in (**I**) (n = 4). (**M** and **N**) Immunohistochemical staining for Ki67 (**M**) and quantitative analysis of Ki67^{+} cells (**N**) in tumor tissues from mice in each group in (**I**). Scale bars, 25 μm (n = 6). (**O**) Analysis of the proliferation of TPC-1 cells treated with or without rhPOSTN for the indicated time by CCK8 assay (n = 3). (**P**) Analysis of the proliferation of TPC-1 cells transfected with POSTN or control vector (n = 6). (**Q** and **R**) Representative images of the immunofluorescence staining of Ki67 (**Q**) and quantification of Ki67^{+} cells (**R**) in TPC-1 cells treated with or without rhPOSTN. Scale bars, 50 μm (n = 5). (**S** and **T**) Representative images of the immunofluorescence staining of Ki67 (**S**) and quantification of Ki67^{+} cells (**T**) in TPC-1 cells transfected with POSTN or control vector. Scale bars, 50 μm (n = 4). Data are shown as means ± SEM. Student's t test (B, D, F, J, L, N, R and T). Two-way ANOVA (O and P). *, $P < 0.05$; **, $P < 0.01$; ***, $P < 0.001$; ****, $P < 0.0001$.

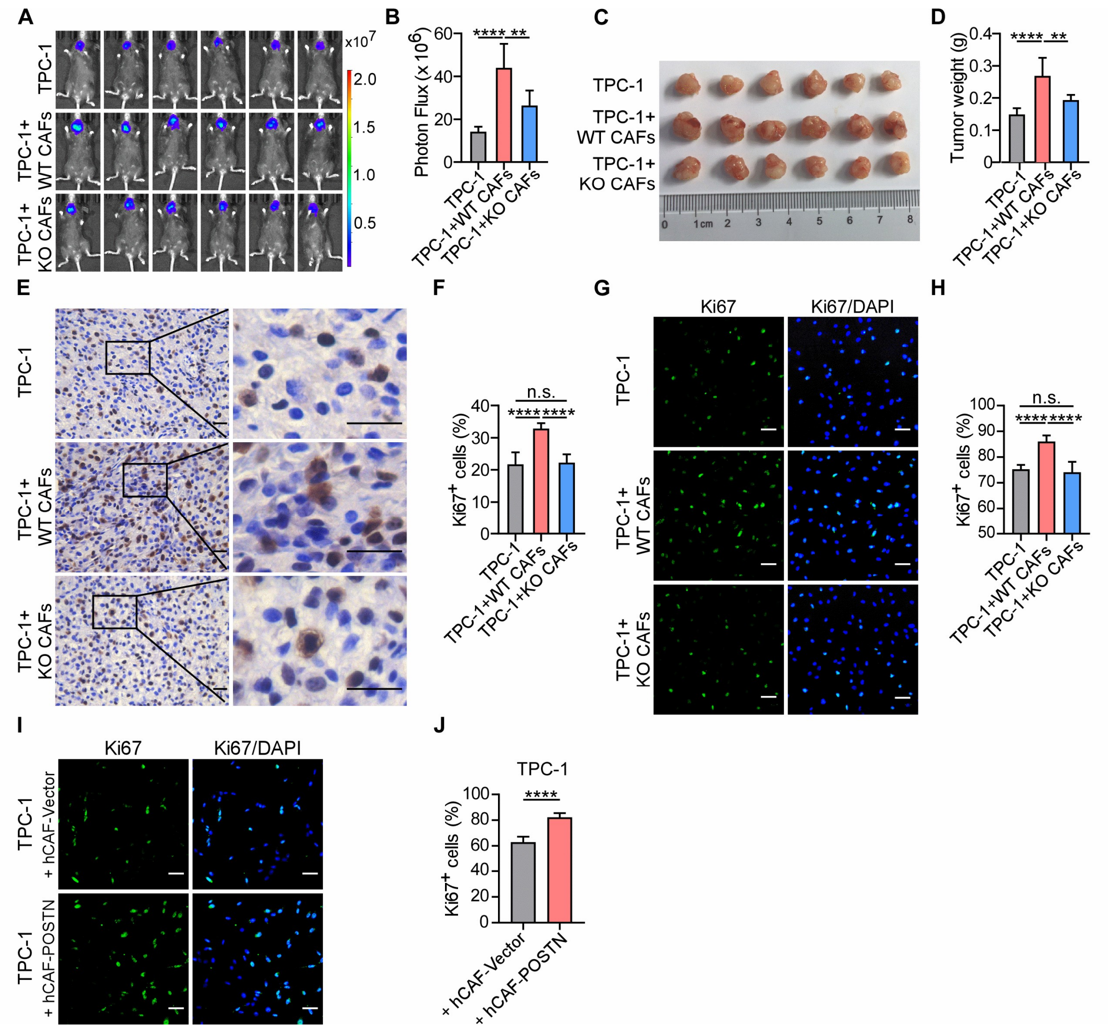

Figure S3. Deficiency of POSTN in CAFs impairs CAF-promoted papillary thyroid tumor growth *in vivo* and TPC-1 cell proliferation *in vitro*. (**A** and **B**) Bioluminescence imaging (**A**) and quantification of tumor burden (**B**) of *Postn*$^{-/-}$*Rag1*$^{-/-}$ mice after orthotopic injection with TPC-1 cells alone or together with WT or KO CAFs (n = 6). (**C** and **D**) Images (**C**) and quantification of weights (**D**) of isolated orthotopic tumors from mice in each group in (**A**) (n = 6). (**E** and **F**) Immunohistochemical staining for Ki67 (**E**) and quantitation of Ki67^{+} cells (**F**) in tumor tissues from each group in (**A**). Scale bars, 25 μm (n = 3). (**G** and **H**) Immunofluorescence images (**G**) and quantitation (**H**) of Ki67^{+} TPC-1 cells cultured alone or co-cultured with WT or KO CAFs (n = 6). (**I** and **J**) Immunofluorescence images (**I**) and quantitation (**J**) of Ki67^{+} TPC-1 cells co-cultured with the vector control or POSTN-overexpressing human CAFs (n = 5). Data are shown as means ± SEM. One-way ANOVA (B, D, F and H). Student's t test (J). **, $P < 0.01$; ****, $P < 0.0001$; n.s., no significant difference.

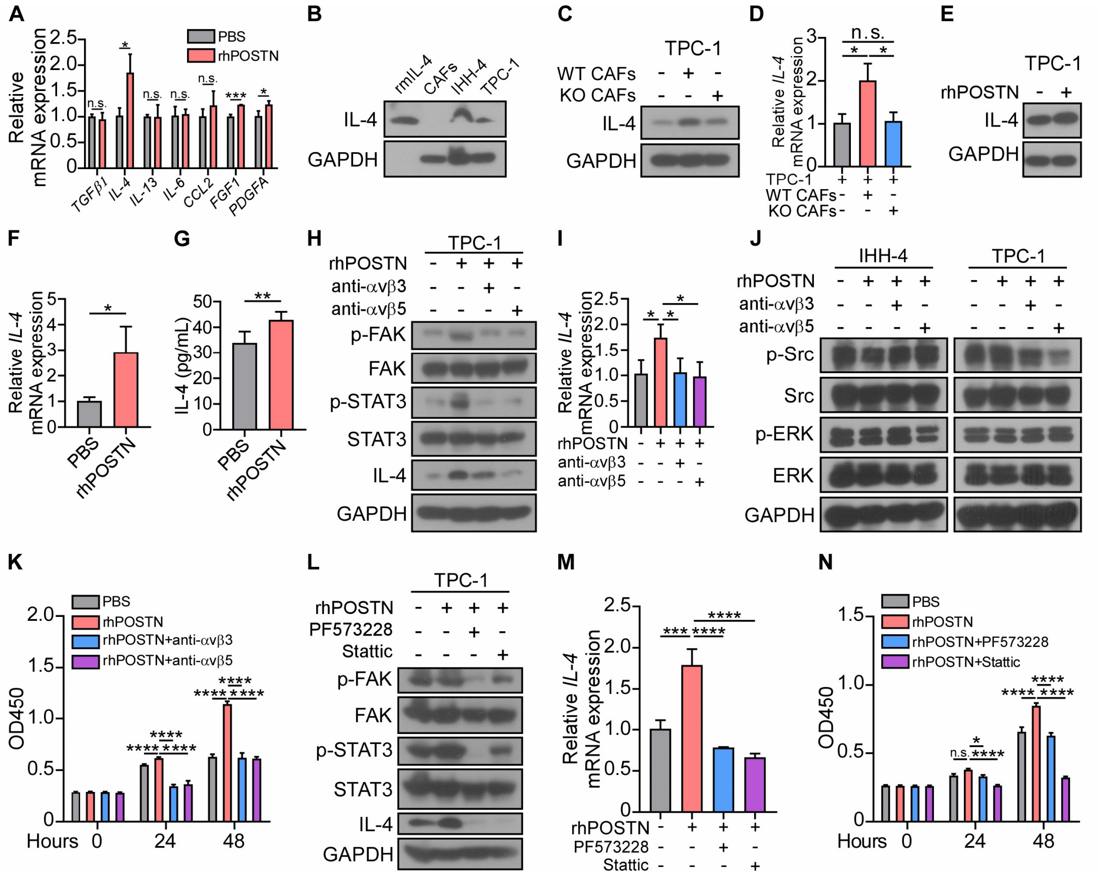

Figure S4. POSTN promotes the proliferation and IL-4 expression in TPC-1 cells by activating the integrin-FAK-STAT3 signaling. (**A**) qRT-PCR analysis of the indicated genes in IHH-4 cells treated with or without rhPOSTN (n = 3). (**B**) Western blot analysis of IL-4 in CAFs, IHH-4 and TPC-1 cells. rmIL-4 was used as a positive control. (**C** and **D**) Western blot (**C**) and qRT-PCR (**D**) analyses of IL-4 expression in TPC-1 cells cultured alone or co-cultured indirectly with WT or KO CAFs (n = 3). (**E-G**) Western blot (**E**), qRT-PCR (**F**) and ELISA (**G**) analyses of IL-4 expression in TPC-1 cells treated with or without rhPOSTN (n = 3). (**H** and **I**) Western blot (**H**) and qRT-PCR (**I**) analyses of the indicated proteins and genes in TPC-1 cells treated with rhPOSTN in combination with antibodies against integrins αvβ3 or αvβ5 (n = 3). (**J**) Western blot analyses of the indicated proteins in IHH-4 or TPC-1 cells treated with rhPOSTN in combination with antibodies against integrins αvβ3 or αvβ5. (**K**) Analysis of the proliferation of TPC-1 cells treated as in (**H**) for the indicated time by CCK8 assay (n = 5). (**L** and **M**) Western blot (**L**) and qRT-PCR (**M**) analyses of the indicated proteins and genes in TPC-1 cells treated with rhPOSTN alone or together with the indicated inhibitors (n = 3). (**N**)

Analysis of the proliferation of TPC-1 cells treated as in (**L**) for the indicated time by CCK8 assay (n = 4). Data are shown as means ± SEM. Two-way ANOVA (A, K and N). One-way ANOVA (D, I and M). Student's t test (F and G). *, $P < 0.05$; **, $P < 0.01$; ***, $P < 0.001$; ****, $P < 0.0001$; n.s., no significant difference.

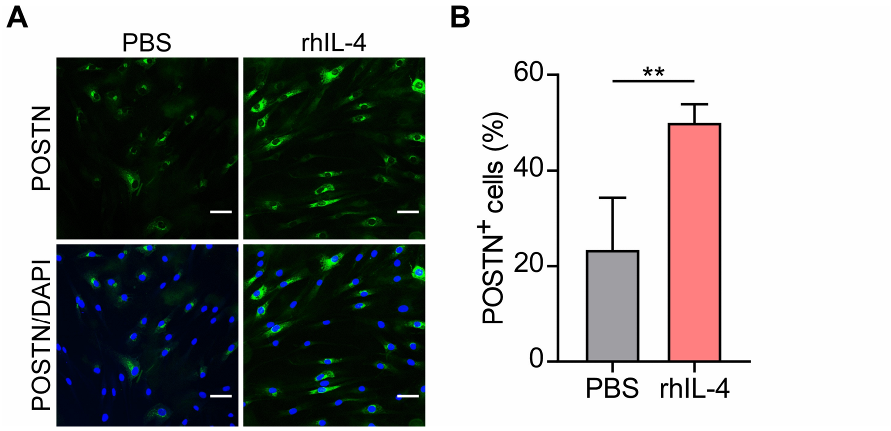

Figure S5. IL-4 promotes POSTN expression in isolated CAFs of human papillary thyroid tumors. (**A** and **B**) Immunofluorescence images (**A**) and quantitation (**B**) of POSTN^{+} cells in isolated CAFs of human papillary thyroid treated with or without rhIL-4. Scale bars, 50 μm (Student's t test, n = 4). Data are shown as means ± SEM. **, $P < 0.01$.

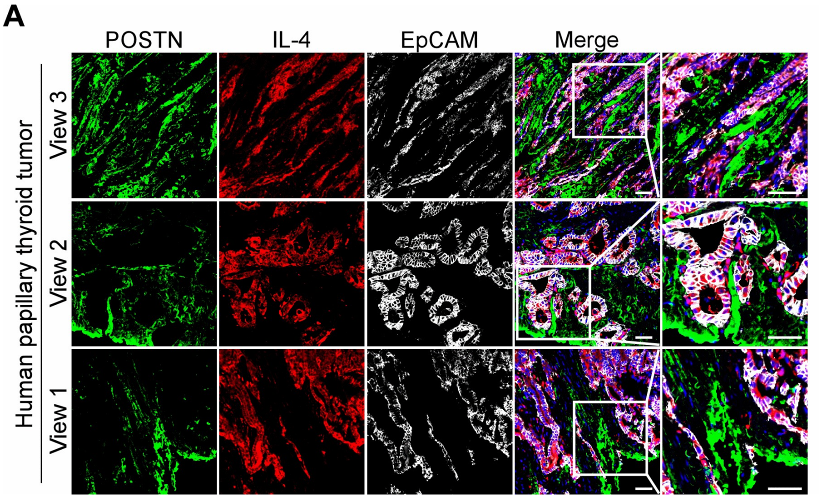

Figure S6. The correlation between POSTN and IL-4 expression in human papillary thyroid tumors. (**A**) Representative images of POSTN, IL-4 and EpCAM in clinical papillary thyroid tumors by multiplex immunofluorescence staining. Scale bars, 50 μm.

Supplementary tables

Table S1. Reagents or Resources used in the experiments

Antibodies	Source	Identifier
Mouse monoclonal anti-Periostin	Adipogen	Cat# AG-20B-0033; RRID: AB_2490231
Rabbit polyclonal anti-α-SMA	Cell Signaling Technology	Cat# 19245; RRID: AB_2734735
Rabbit monoclonal anti-EpCAM	Abcam	Cat# ab213500; RRID: AB_2884975
Rabbit polyclonal anti-Ki67	Abcam	Cat# ab15580; RRID: AB_443209
Rabbit monoclonal anti-IL-4	Abcam	Cat# ab300138; RRID: N/A
Rabbit monoclonal anti-Phospho-STAT3	Cell Signaling Technology	Cat# 9145; RRID: AB_2491009
Rabbit monoclonal anti-GAPDH	Cell Signaling Technology	Cat# 92310; RRID: N/A
Rabbit monoclonal anti-Cyclin D1	Cell Signaling Technology	Cat# 2978; RRID: AB_2259616
Rabbit monoclonal anti-β-actin	Cell Signaling Technology	Cat# 8457; RRID: AB_10950489
Rabbit monoclonal anti-IL-4	Abcam	Cat# ab62351; RRID: N/A
Rabbit polyclonal anti-IL-4	ABclonal	Cat# A5649; RRID: AB_2766409
Rabbit polyclonal anti-Phospho-FAK	Cell Signaling Technology	Cat# 3283; RRID: AB_2173659
Rabbit monoclonal anti-FAK	Abcam	Cat# ab40794; RRID: AB_732300
Mouse monoclonal anti-STAT3	Cell Signaling Technology	Cat# 9139; RRID: AB_331757
Rabbit monoclonal anti-Phospho-ERK1/2	Cell Signaling Technology	Cat# 9101; RRID: AB_331646
Rabbit monoclonal anti-ERK1/2	Cell Signaling Technology	Cat# 9102; RRID: AB_330744
Rabbit monoclonal anti-Phospho-SRC	Cell Signaling Technology	Cat# 2101; RRID: AB_331697
Rabbit monoclonal anti-SRC	Cell Signaling Technology	Cat# 2109; RRID: AB_2106059
Rabbit monoclonal anti-Phospho-STAT6	Abcam	Cat# ab188080; RRID: AB_2862613
Rabbit monoclonal anti-STAT6	ABclonal	Cat# A19120; RRID: N/A
Rabbit monoclonal anti-CD31	Abcam	Cat# ab76533; RRID: AB_1523298
Anti-Integrin αvβ3 antibody	Millipore/Merck	Cat# MAB1876-Z; RRID: AB_569486
Anti-Integrin αvβ5 antibody	Millipore/Merck	Cat# MAB1961; RRID: AB_94466
Rabbit monoclonal anti-F4/80	Abcam	Cat# ab300421; RRID: AB_2936298
Rabbit monoclonal anti-IL-4R	Abcam	Cat# ab271041; RRID: N/A
Rabbit monoclonal anti-Phospho-STAT6	Abcam	Cat# ab235591; RRID: N/A
Rabbit IgG antibody	Sigma-Aldrich	Cat# I5006; RRID: AB_1163659
**Chemicals, peptides, and recombinant proteins**		
FAK inhibitor PF573228	Selleck Chemicals	Cat# S2013
STAT3 inhibitor Stattic	Selleck Chemicals	Cat# S7024
Recombinant Human Periostin Protein	R&D Systems	Cat# 3548-F2-050
Recombinant Mouse IL-4 Protein	ABclonal	Cat# RP01161
Recombinant Human IL-4 Protein	ABclonal	Cat# RP00995

AS1517499	MedChemExpress	Cat# HY-100614
Collagenase II	Gibco	Cat# 17101015
DNase I	Roche	Cat# 04716728001
**Critical Commercial Assays**		
Mouse Periostin/OSF-2 DuoSet ELISA	R&D	Cat# DY2955
Human IL-4 ELISA Kit	ABclonal	Cat# RK00003
Cell Counting Kit-8	Beyotime	Cat# C0038
TriPure Isolation Reagent	Roche	Cat# 11667165001
ReverTra Ace qPCR RT kit	TOYOBO	Cat# FSQ-101
BrightCycle Universal SYBR Green qPCR Mix with UDG	Abclonal	Cat# RK21219
**Experimental Models: Organisms**		
Mouse: B6.129-*Postn*^{tm1Jmol}/J	The Jackson Laboratory	JAX: 009067
Mouse: C57BL/6	The Animal Laboratory Center of Xiamen University	N/A
Mouse: B6.129S7-*Rag1*^{tm1Mom}/J	The Jackson Laboratory	JAX: 002216

Table S2. Primer sequences for FISH

**FISH probe**	Sequence (5'-3')
FAM-*Postn* Mouse	GCGTTGGTCCATGCTCAGAGTGTC
TAMRA-*Acta2* Mouse	AGAGCTACGAACTGCCTGAC
TAMRA-*POSTN* Human	GCTACCACGAACAAACTTAATTTGGATG
FAM-*ACTA2* Human	GAGACAGCACCGCCTGGATAGCC

Table S3. Primers of plasmids for luciferase reporting assay

**Gene**	Vector	Primer sequence 5'	Primer sequence 3'
Postn promotor Mouse	pGL3-basic	TCTTACGCGTGCTAGCCCTCATCAATAAACATTCTAAC	GATCGCAGATCTCGAGAGCAGACTGCTCTGAGAACT
Stat6 Mouse	pCMV5	CCGGAATTCATGTCTCTGTGGGGCCTAATTTC	CTAGTCTAGATCACCAGCTGGGGTTGGTC
Postn promotor Mut1 Mouse	pGL3-basic	CCCGGGCTCGAGTTCCAACTGTAAAACAGCCACAGT	CGCAGATCTCGAGAGCAGACTGCTCTGAGAACTCAGA
Postn promotor Mut2 Mouse	pGL3-basic	CTAGCCCGGGCTCGAGACCCTGCAAATGCCAACAGT	CGCAGATCTCGAGAGCAGACTGCTCTGAGAACTCAGA

Table S4. Primers of *Postn* promoter segments for ChIP-qPCR assay

**Promoter region**		Sequence (5'-3')
Postn (-1643~-1439bp)	Forward	CCTCATCAATAAACATTCTAACAGCC

	Reverse	TCTCTTATTTATATTTATCTAAATGAAA
Postn (-1417~-1164bp)	Forward	GCCCTGGAAGGAACATAGTTTACT
	Reverse	TTAGTCCAGAGAGAATTCCTTATCTGG
Postn (-1144~-892bp)	Forward	TTCCAACTGTAAAACAGCCACAGT
	Reverse	GGTCTTCACACATGACCAGATATGA
Postn (-867~-617bp)	Forward	AAACAAAAAAACAAAGTGTCTTCCC
	Reverse	ATCTATCTATCTATCTATCTATCTTTC
Postn (-563~-272bp)	Forward	AGATCTTTGTTACTTAACCCTCTATAC
	Reverse	ATTCTGAGTTTAGGGATCTCTCTGCC
Postn (-133~+123bp)	Forward	AGGCCATCGCAAGCTTCAG
	Reverse	CCTGATCCCGACCCCTGA
Postn (+157~+444bp)	Forward	CACTTAGCTTGTTCTAGAAATATGCGT
	Reverse	AAAATGCCAAGTAAACTGAATTAGTAA
Postn (+517~+750bp)	Forward	GTTTTAAGATGTCCTGCAAGATATCAG
	Reverse	CTGCTCTGAGAACTCAGAAAAATGA

Table S5. Sequences of primers for qRT-PCR analysis

Gene		Sequence (5'-3')
POSTN	Forward	GCACTCTGGGCATCGTGGGA
Human	Reverse	AATCCAAGTTGTCCCAAGCC
GADPH	Forward	AGGTCGGAGTCAACGGATTTG
Human	Reverse	GGGGTCATTGATGGCAACA
IL-4	Forward	CGGCAACTTTGTCCACGGA
Human	Reverse	TCTGTTACGGTCAACTCGGTG
TGFβ1	Forward	CGCCGAGCCCTGGACACCAACT
Human	Reverse	TGCGTGTCCAGGCTCCAAATGTAGG
IL-13	Forward	CCTCATGGCGCTTTTGTTGAC
Human	Reverse	TCTGGTTCTGGGTGATGTTGA
IL-6	Forward	AGACAGCCACTCACCTCTTCAG
Human	Reverse	TTCTGCCAGTGCCTCTTTGCTG
CCL2	Forward	AGAATCACCAGCAGCAAGTGTCC
Human	Reverse	TCCTGAACCCACTTCTGCTTGG
FGF1	Forward	ACACCGACGGGCTTTTATACG
Human	Reverse	CCCATTCTTCTTGAGGCCAAC
PDGFA	Forward	GCAAGACCAGGACGGTCATTT
Human	Reverse	GGCACTTGACACTGCTCGT
Postn	Forward	TAGCCCAATTAGGCTTGGCATCC

Mouse	Reverse	TAAGAAGGCGTTGGTCCATGCT
Gadph	Forward	TGTGTCCGTCGTGGATCTGA
Mouse	Reverse	TTGCTGTTGAAGTCGCAGGAG
Il-4	Forward	ATCATCGGCATTTTGAACGAGGTC
Mouse	Reverse	ACCTTGGAAGCCCTACAGACGA
Ccnd1	Forward	GCAGAAGGAGATTGTGCCATCC
Mouse	Reverse	AGGAAGCGGTCCAGGTAGTTCA
Acta2	Forward	ATGCTCCCAGGGCTGTTTTCCCAT
Mouse	Reverse	GTGGTGCCAGATCTTTTCCATGTCG
